# Revisiting traditional SSR based methodologies available for elephant genetic studies

**DOI:** 10.1038/s41598-021-88034-9

**Published:** 2021-04-22

**Authors:** M. S. L. R. P. Marasinghe, R. M. R. Nilanthi, H. A. B. M. Hathurusinghe, M. G. C. Sooriyabandara, C. H. W. M. R. B. Chandrasekara, K. A. N. C. Jayawardana, M. M. Kodagoda, R. C. Rajapakse, P. C. G. Bandaranayake

**Affiliations:** 1 Department of Wildlife Conservation, 811/A, Jayanthipura Road, Battaramulla, 10120 Sri Lanka; 2 Agricultural Biotechnology Centre, Faculty of Agriculture, University of Peradeniya, Peradeniya, 20400 Sri Lanka; 3 Department of National Zoological Gardens, Anagarika Dharmapala Mawatha, Dehiwala, 10350 Sri Lanka

**Keywords:** Biological techniques, Genetic techniques, Biodiversity, Molecular ecology

## Abstract

Asian elephant (*Elephas maximus*) plays a significant role in natural ecosystems and it is considered as an endangered animal. Molecular genetics studies on elephants’ dates back to 1990s. Microsatellite markers have been the preferred choice and have played a major role in ecological, evolutionary and conservation research on elephants over the past 20 years. However, technical constraints especially related to the specificity of traditionally developed microsatellite markers have brought to question their application, specifically when degraded samples are utilized for analysis. Therefore, we analyzed the specificity of 24 sets of microsatellite markers frequently used for elephant molecular work. Comparative wet lab analysis was done with blood and dung DNA in parallel with in silico work. Our data suggest cross-amplification of unspecific products when field-collected dung samples are utilized in assays. The necessity of Asian elephant specific set of microsatellites and or better molecular techniques are highlighted.

## Introduction

Elephants are the largest living land mammals on the earth and they play an important role in ecosystems. Other than the large body size, they are distinct from other animals having long muscular trunks and tusks^1^. Further, the elephants are famous for their extraordinary sensory system and chemical communication^2^, unique vocal communication^3–5^, memory power and intelligence^6–9^ and social behavior^5,10,11^.

Elephants belong to the family Elephantidae and the other Proboscidae evolved over 60 million years ago^12^. There are two elephant species; the Asian elephant, *Elephas maximus* and the African elephant, *Loxodonta africana*^1, 13^. Nevertheless, some morphological and molecular evidences suggest, African elephants are not one species but, two. The Savannah elephant *L. africana africana*^14^ and forest elephant *L. africana cyclotis*^13,15–20^ are deeply divergent lineages separated about 4–7 million years ago^17,21^. It is also recognized that there are three subspecies of Asian elephant; *Elephas maximus maximus* from Sri Lanka (1758), *Elephas maximus indicus* from Asian mainland/India (1978) and *Elephas maximus sumatranus* from Sumatra (1847)^22–24^.

Historical evidences suggest that the Asian elephant ranged from Mesopotamia in the west to the South and Southeast Asia, China and to the Yangtze River^24–30^. However, currently it exists on about 15% of the historical range, in a fragmented and isolated population in South and Southeast Asia^24,30^. Further, *E. maximus* is classified as an ‘endangered’ species in IUCN (International Union for Conservation of Nature) red list^31,32^. Nevertheless, Sri Lanka is the homage of a considerable *E. maximus* population consisting of about 5000–6000 elephants (DWC records 2011).

The molecular work on elephant started in the early 1990s and initial studies focused on a few mitochondrial regions^28–30,33–39^, nuclear genes and microsatellites or simple sequence repeats (SSR)^20,40–55^. The recent studies include techniques such as multiplex genotyping^44^, protein electrophoresis^56^, single nucleotide polymorphisms (SNPs)^57,58^, shotgun and restriction site-associated DNA sequencing (RAD-seq)^57,58^ and transcriptomics^59–61^. Further, relatively high coverage ~ 33.4X Savannah African elephant genome^21^ and ~ 94.4X of Asian elephant genome^62^ are also available now. Scientists have also studied the phylogenetic relationship between *E. maximus, L.africana* and *Mammuthus primigenius* (woolly mammoth)^21,63,64^.

However, thus far SSR is the most widely used technology in elephant molecular analysis, for resolving phylogenetic relationships^12, 21,39,63–66^, population structure and social organization^5,37,41, 67–71^, illegal poaching and ivory trade^19,72–76^, ecology and conservation^23,27,28,77–79^, distribution and behavior^24^ and sex determination^73,77,80–82^. While the early SSR studies were based on PCR amplification followed by polyacrylamide gel electrophoresis and silver staining^55,83^, later studies utilized relatively advanced technologies such as fragment analysis or capillary electrophoresis^19,40,52,84–86^. Elephant blood or tissues, as well as dung, have been utilized in the previous studies^37,87,88^. Interestingly, the SSRs developed for the African elephant have been successfully utilized for Asian elephant studies^50,52,54,89^ and vice versa^19,90^. When the first SSR study on Asian elephant was conducted in 2001, neither the human genome nor the elephant genome was available. Therefore, the specificity of SSRs and the possibility of amplification from other animals, plants and fungal species were not tested. Interestingly, the same set of SSR is still being used around the world^84,86,91,92^. Nevertheless, the specificity of recently developed SSRs was tested in silico using available genomic data and only the specific ones were selected for subsequent lab work^84^. The specificity is very critical when the wild-collected dung samples are the only available material for molecular studies. Here we report lessons learned while utilizing previously published SSRs for amplification of blood DNA from identified elephants and dung DNA extracted from wild-collected samples from Sri Lankan forests.

## Results

### Optimization of DNA extraction protocol

The quality and quantity of DNA are the key factors to the success of any downstream application. As expected, quality DNA was obtained from the elephant blood using a commercially available kit. However, the commercial kit QIAGEN QIAamp Fast DNA Stool Mini Kit (Cat.No, 51,604) protocol that is optimized for faecal samples had to be modified to extract good quality DNA from elephant dung. From the studied temperature and time combinations, 56 °C for 4 h incubation resulted in high-quality DNA with the least PCR inhibitors and therefore, was selected for dung DNA extraction from the rest of the samples (Supplementary Figure S1A). The optimized protocol worked efficiently for fresh dung less than 24 h old, and for the outermost mucus layer stored at 4 °C for 2 weeks (Supplementary Figure S1B).

### Optimization of PCR conditions for elephant blood DNA

Among the tested reaction and thermocycler conditions, the optimum PCR mixture consisted of 1 × GoTaq Flexi buffer (Promega, Cat No: M891A), 160 µM dNTP mixture (Promega, Cat No: U151B), 1.5 mM MgCl_2_ (Promega, Cat No: A35H), 0.12 M Spermidine, 0.8% PVP, 0.2 µM Reverse Primer, 0.2 µM FAM-labeled M13 Forward Primer, 0.08 µM M13 tailed Forward primer (Integrated DNA Technologies IDT, Singapore), 1 Unit Go Taq Flexi DNA polymerase (Promega, Cat No: M8295) and 100 ng RNase treated DNA template in a 25 µL reaction volume. The touchdown PCR program consisted of initial denaturation at 94 °C for 5 min, followed by 30 cycles of 94 °C for 1 min, 58 °C for 30 s and 72 °C for 1 min and 08 cycles of 94 °C for 1 min, 55 °C for 30 s and 72 °C for 1 min and final extension at 72 °C for 5 min.

### Selection of SSRs

**Figure 1:**
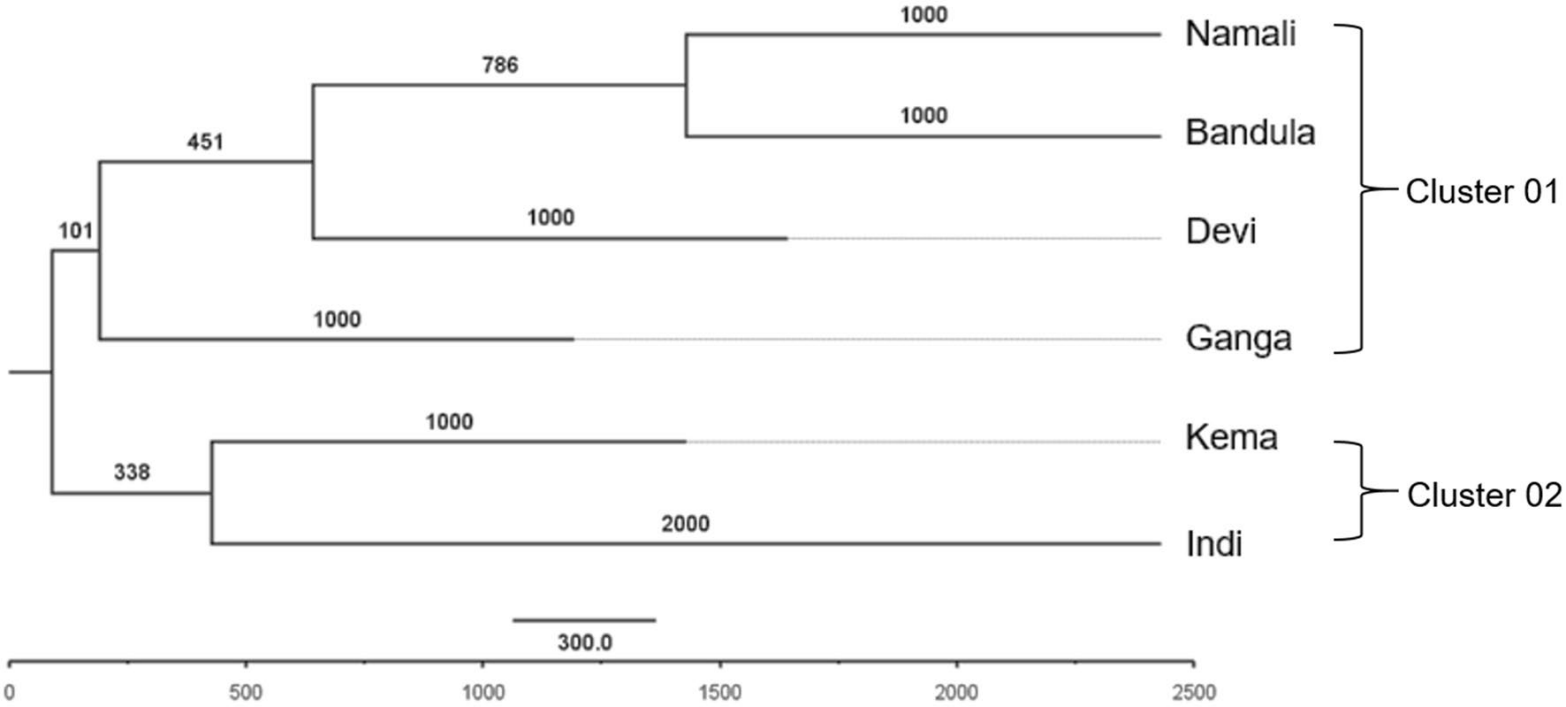
Relationship among Asian elephants in the National Zoological Garden, Sri Lanka. The phylogenetic tree developed based on the binary data matrix obtained by reading distinctive peaks of fragment analysis data, using UPGMA method. Bootstrap probability values for 1000 bootstrap resamples are indicated at each node.

First, we selected the ten most polymorphic SSRs from previous studies to assess their suitability for studying the genetic relationship of the current Sri Lankan elephant population. For this, we selected six elephants housed in the National Zoological Garden of Sri Lanka. This could be considered as a representative sample of Sri Lankan elephants since they have different morphological features and were from different geographical locations. The blood DNA extracted amplified with ten SSRs resulted di, tri and tetra-nucleotide repeats. However, of the ten markers, only eight were polymorphic (Table 1). The EMU17 presented the highest number of alleles (Table 1), while both EMU10 and EMU13 amplified only two alleles. The UPGMA dendrogram built with capillary sequencing data (Supplementary Figure S2) showed the relationship among the six elephants in the National Zoological Garden (Fig. 1).

**Table 1:** Summary of capillary electrophoresis data.

No	Locus	Reported allele size from literature (bp)	Allele size reported in the current study (bp)	Reported number of alleles from literature	Alleles observed in the current study			
					Blood (06 samples)		Dung (04 samples)	
					Total no: of alleles observed	Polymorphic percentage (%)	Total no: of alleles observed	Polymorphic percentage (%)
1	LafMS02	136–168	124–181	8	–		10	100.0
2	LafMS06	138–156	138–185	8	–		6	50.0
3	EMX-1	137–152	133–222	4	–		5	80.0
4	EMX-2	217–223	165–242	2	–		6	33.33
5	EMX-3	238–254	238–273	2	–		4	100.0
6	EMX-4	351–387	351–400	3	–		3	100.0
7	EMX-5	248–263	202–281	3	–		7	100.0
8	LA2	226–241	217–364	4	–		6	66.67
9	LA3	166–172	127–204	3	–		7	71.42
10	LA4	111–137	111–137	4	–		2	100.0
11	LA5	130–154	140–160	2	–		3	33.33
12	LA6	155–214	161–185	3	–		8	100.0
13	EMU03	137–143	106–162	4	–		6	100.0
14	EMU04	97–107	115–173	6	6	83.33	9	100.0
15	EMU06	146–158	122–164	4	5*	80.0	4	75.0
16	EMU07	102–122	111–200	5	3	100.0	8	100.0
17	EMU09	163–169	140–185	4	4	75.0	5	100.0
18	EMU10	94–104	106–145	5	2	0	6	100.0
19	EMU11	122–136	137–149	5	4	75.0	6	100.0
20	EMU12	120–152	110–155	5	6	66.67	6	83.33
21	EMU13	100–110	114–171	6	2	0	5	100.0
22	EMU14	130–138	143–165	4	–	–	8	87.5
23	EMU15	142–154	131–170	6	3	100.0	7	100.0
24	EMU17	119–137	108–149	8	7	100.0	3	100.0

The blood and dung samples are not from the same elephants. However, monomorphic bands were present in all the cases.

*For some loci only few samples amplified (3 out of 6).

(–) Not tested in the current study.

The six individuals were grouped into two clusters, where *Namalee, Bandula, Devi* and *Ganga* clustered together while *Kema* and *Indi* were together. Interestingly, *Bandula* and *Namalee* have similar morphological features, such as, the abundance of freckles (depigmentation) on the trunk (Supplementary Figure S3a,b respectively). *Devi* and *Ganga* have an average number of freckles on the trunk (Supplementary Figure S3c,d respectively). The tusker, *Kema* grouped separately with *Indi* and both do not have freckles on the trunk (Supplementary Figure S3e,f).

### Selection of polymorphic SSR loci for Sri Lankan wild elephant analysis

We initially selected ten most polymorphic SSRs from previous studies and assessed the suitability of them using blood DNA extracted from a representative group of elephants. Since only eight of them were polymorphic and number of alleles observed were less than previous studies, we included 24 SSRs identified from the literature. Further, we selected another set of elephants including one animal with Indian origin to address the possibility of less overall genetic diversity among elephants in National Zoological Garden of Sri Lanka. At this point, we also moved from blood to dung, since collecting blood from a large number of wild elephants are not practically possible for any study. Therefore, dung samples from four randomly selected domesticated elephants were included in this experiment.

**Figure 2:**
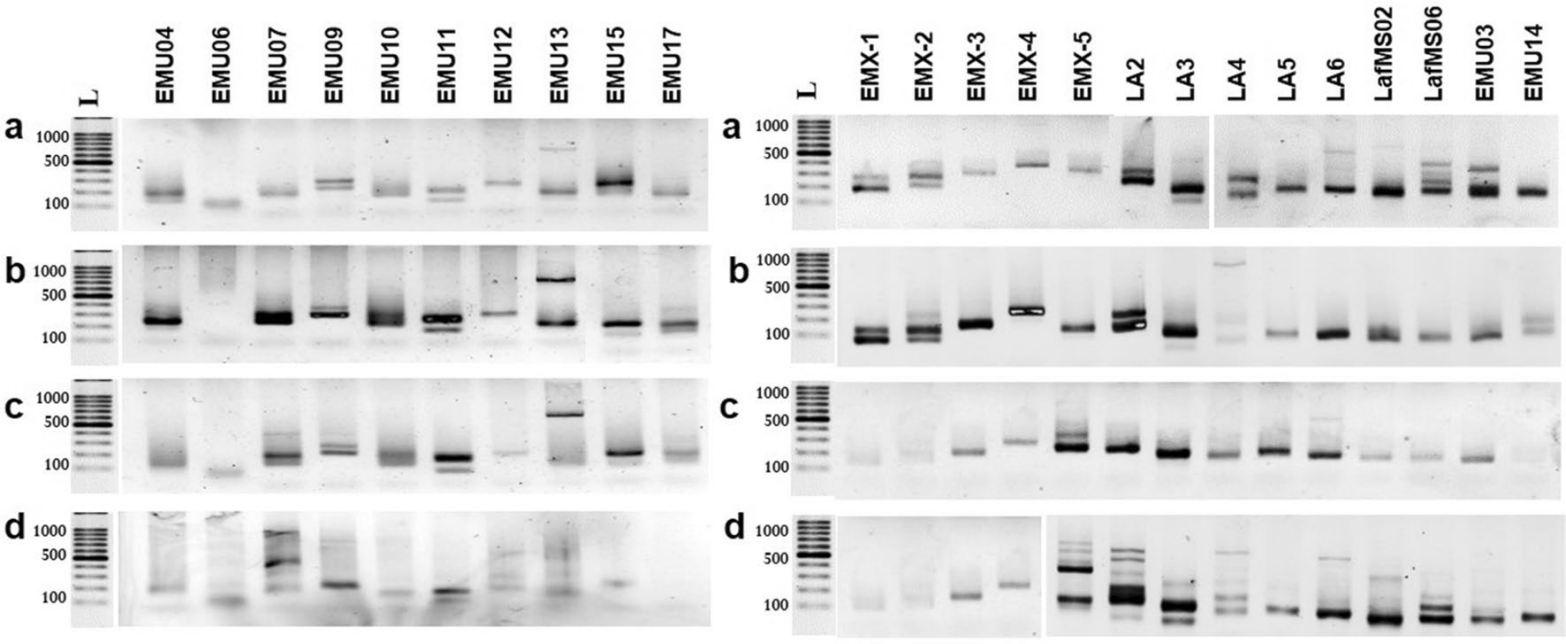
Identification of polymorphic markers for amplification of dung DNA. Primer names are given on the top of each well. L-100 bp ladder (Promega, Cat no: G2101), (**a**) – (**c**) Sri Lankan origin, (**d**) Indian origin. Full-length gels are presented in Supplementary Figure S4.

Of the multiple bands detected in 1.5% Agarose gel (Fig. 2), some were approximately similar to the previously reported work and for the bands resulted from blood samples in the current study, while the others were much bigger. Therefore, only the fragments with the expected size were considered in the analysis (Table 1). Except for LafMS06, LA4, EMU13 and EMU17, all the other SSRs showed more alleles than previous reports. Nine out of ten SSRs used for blood DNA work, amplified more alleles when those were used for amplification from dung (Table 1). The EMU06 had interesting amplification even in the blood where only 50% (3 out of 6) of samples resulted visible products. Though the blood and dung were not from the same elephants, monomorphic bands were consistently present in all the samples. Further, numbers of bands were consistently higher in dung samples. Though one of the elephants used in dung analysis is with Indian origin, no clear differences were observed in amplification patterns (Fig. 2d). The current analysis also showed that the primers designed for African elephants are applicable for Asian elephants having both Sri Lankan and Indian origin. Of the 24 SSRs, LafMS02 amplified the highest total number of alleles, ten (Table 1), with no obvious multiple amplifications on gels (Fig. 2). Therefore, LafMS02 ranked top among tested SSRs was selected for analysis of selected Sri Lankan wild elephant population.

### Analysis of selected wild populations using LafMS02

**Figure 3:**
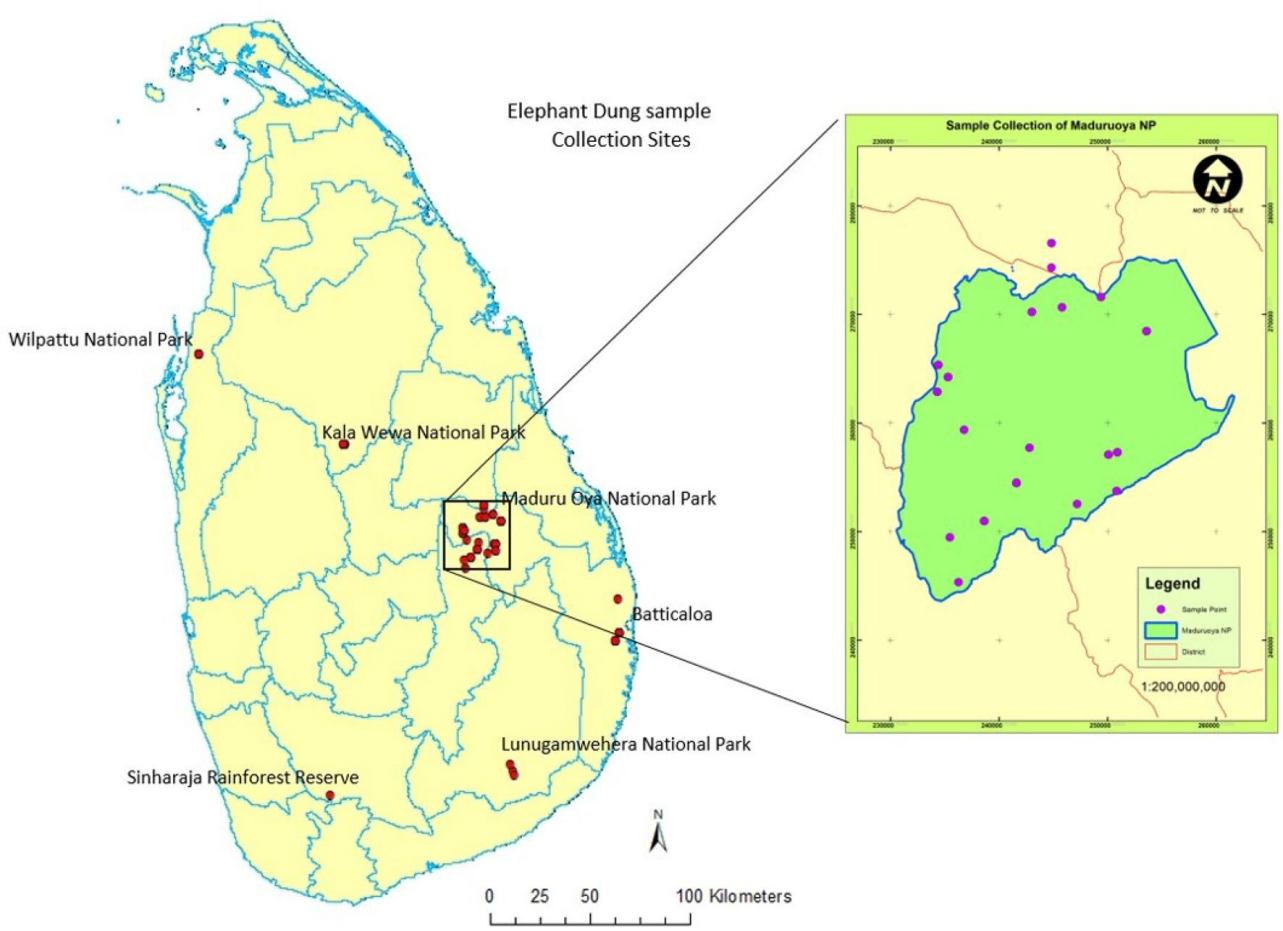
Distribution of wild elephant dung sampling sites in Sri Lanka. The number of dung samples collected varied and decided in proportion to the number of elephants in a considered population. A total of 93 samples were collected covering most of the National Parks in Sri Lanka from wild elephants.

**Figure 4:**
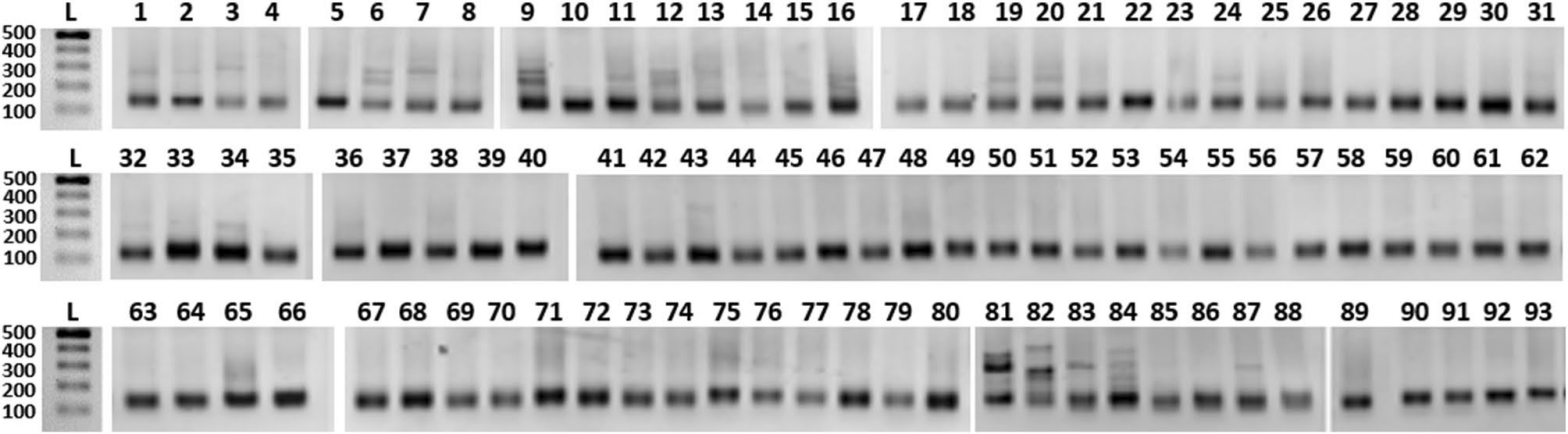
Amplification with SSR—LafMS02. L-100 bp ladder (Promega Cat no: G2101), 1–93 dung DNA samples collected from wild elephants. Full-length gels are presented in Supplementary Figure S5.

A total of 93 wild elephant dung DNA samples collected from different wildlife parks in Sri Lanka (Fig. 3) were amplified with LafMS02 primers. There were obvious multiple amplifications, representing different sizes in some samples, for example, samples 09, 81, 82, 83 and 84 (Fig. 4). This observation led us to further test the specificity of the selected SSRs.

**Figure 5:**
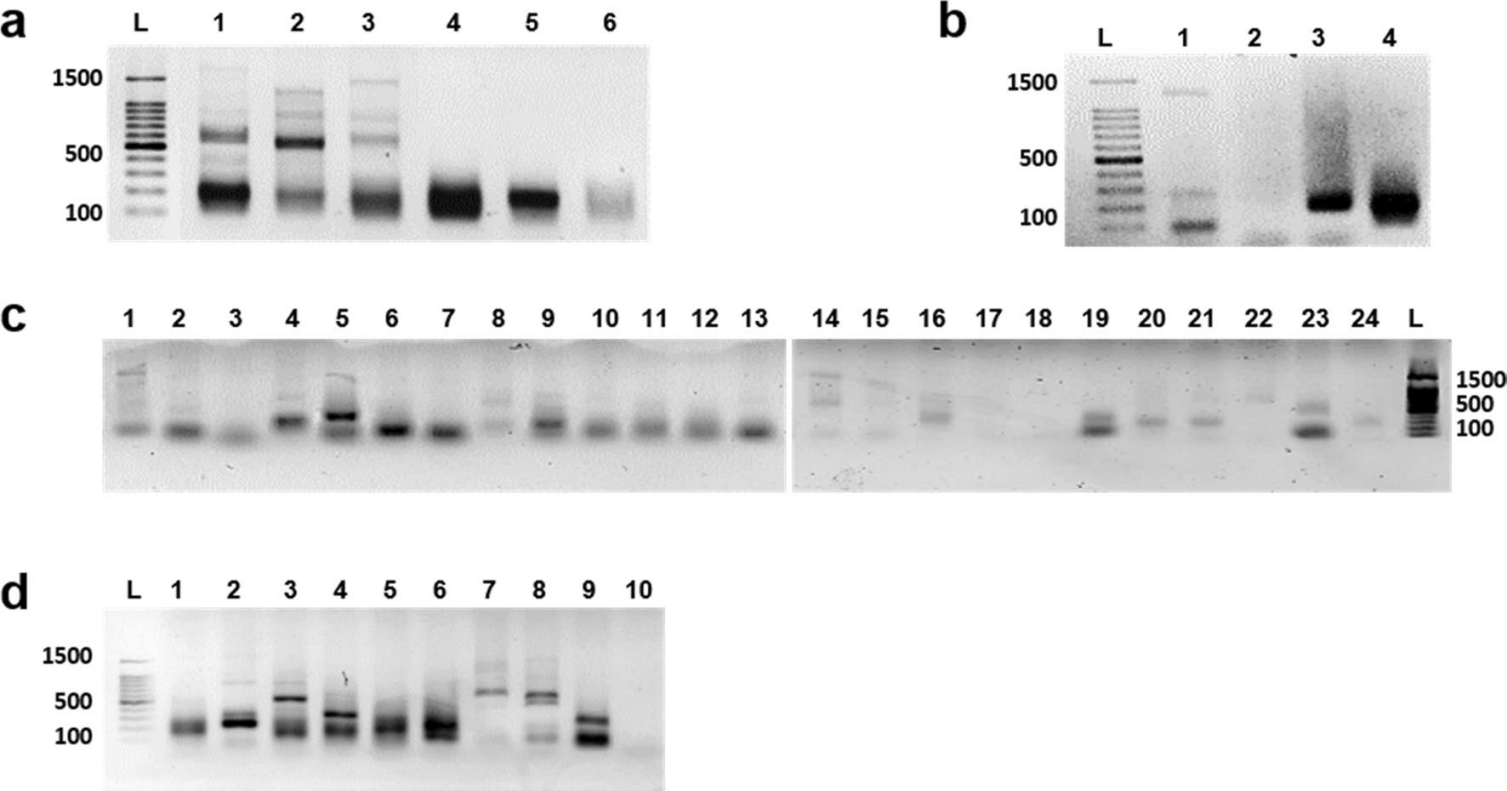
Testing the specificity of SSR primers. (**a**) Amplification of LafMS02 primer. L-100 bp molecular weight marker 1: Elephant dung DNA (from the mucus layer) 2: Fibers taken from inside of the bolus 3: Fibers taken from multiple samples 4: Mixture of Plant DNA (*C. zeylanicum*, *O. rhizomatis*, *S. album* and *S. zeylanica*) 5: Elephant Blood DNA (from elephant no: 66) 6: Negative control-water. (**b**) Amplification of EMU14 primer. L-100 bp molecular weight marker 1: Elephant dung DNA (from the mucus layer), 2: Fibers taken from inside of the bolus 3: Mixture of Plant DNA (*C. zeylanicum*, *O. rhizomatis*, *S. album* and *S. zeylanica*), 4: Elephant Blood DNA (from elephant no: 66). (**c**) Amplification of plant DNA with 24 primers.1: EMU03, 2: EMU04, 3: EMU06, 4: EMU07, 5: EMU10, 6: EMU09, 7: EMU11, 8: EMU12, 9: EMU13, 10: EMU14, 11: EMU15, 12: EMU17, 13: LafMS02, 14: LafMS06, 15: LA2, 16: LA3, 17: LA4, 18: LA5, 19: LA6, 20: EMX-1, 21: EMX-2, 22: EMX-3, 23: EMX-4, 24: EMX-5, L-100 bp ladder. (**d**) Amplification of plant DNA with nine selected primers (repeated PCR). L-100 bp ladder 1: EMU07, 2: EMU09, 3: EMU10, 4: EMU13, 5: EMU14, 6: EMU15, 7: LafMS06, 8: EMX-4, 9:LA6, 10: Negative control (water). Full-length gels are presented in Supplementary Figure S6.

For that, first, we used LafMS02 primers to amplify DNA from blood and dung from the same wild elephant, a sample inside of the dung bolus, a mixture of inner layers of dung bolus and a mixture of plant DNA (Fig. 5a). The plant mixture included equal amounts of DNA from *Cinnamomum zeylanicum, Oryza rhizomatis, Santalum album* and *Strobilanthes zeylanica*. Aclear single band appeared on the gel from blood DNA while multiple bands were resulted from the outermost mucus layer of a dung bolus of the same elephant. Interestingly, plant DNA mixture also resulted approximately similar size band to that of the blood DNA. Further, similar size products were detectable in the DNA extracted from the inner fibrous layer of the dung bolus as well as with a mixture of fibrous layers from different dung bolus. To further verify the results, the same analysis was done with another set of primers, EMU14, selected from the list. Results were similar for having multiple amplifications in dung DNA and parallel size products amplified from blood and plant DNA (Fig. 5b).

### Amplification from plant DNA

The results above directed us to test whether such cross-amplification exists in other SSRs selected from previous studies. The same plant DNA mixture was used as the template with the other 24 primers sets selected (Fig. 5c). The PCR was repeated for eight selected primers from above and products were separated on a 2.0% agarose gel (Fig. 5d). Out of 24, only three SSRs, EMU06, LA4 and LA5 did not show clear amplification. PCR of plant DNA mixture also resulted in bands of similar size as that from blood DNA.

### Increasing annealing temperature to increase the specificity

**Figure 6:**
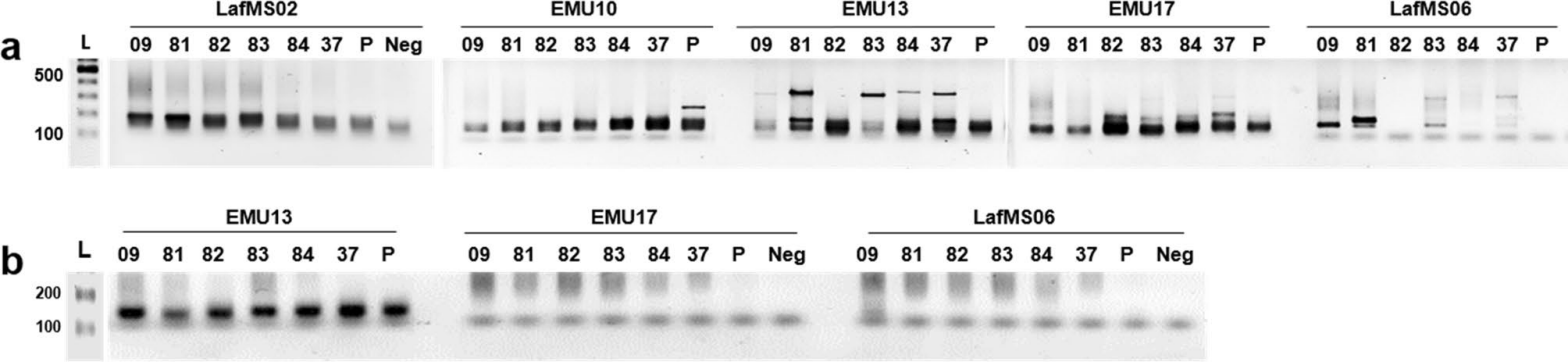
Optimization of annealing temperature. (**a**) Annealing temperatures 62–58 °C in ‘touchdown 58’ PCR for randomly selected samples (09,81,82,83,37) using five polymorphic primers; LafMS02, EMU10, EMU13, EMU17, LafMS06), L-100 bp ladder (Promega, Cat no: G2101), P—Plant DNA mixture, N—Negative control (water). (**b**) Annealing temperatures 69–65 °C in ‘touchdown 65’ PCR for three primers having high annealing temperature for randomly selected samples (09,81,82,83,84,37), L-100 bp ladder (Promega, Cat no: G2101), P—Plant DNA mixture, N—Negative control (water). Full-length gels are presented in Supplementary Figure S7.

It is suggested to use higher annealing temperatures to avoid unspecific amplifications^93^. The majority of selected primers had the melting temperature of 58 °C while few had little higher or lower temperatures (Supplementary Table S1). We used a touchdown PCR algorithm to increase the specificity of primer binding. Nevertheless, higher annealing temperatures of 62–58 °C and 69–65 °C were tested for a selected set of primers (Fig. 6a,b). Increasing the annealing temperature to 62 °C reduced the intensity of the expected products, for example in EMU17 and LafMS06. However, there was no clear change in the intensity of unexpected amplifications, without affecting the expected product. All three primer sets; EMU13, EMU17 and LafMS06 resulted clear multiple amplifications even at 62 °C. However, increasing the annealing temperature to 69 °C affected amplification in all three primers tested (Fig. 6b). These results suggest that increasing annealing temperature does not help to get rid of cross-amplification of contaminating plant DNA possibly co-extracted with elephant DNA.

### Cross-amplification from human DNA

Human DNA contamination is also a possibility of assessing the degraded DNA^84^. Therefore, twelve selected SSRs from the list were assessed with human blood DNA, plant DNA mixture together with three elephant dung DNA samples in three different annealing temperatures 58 °C, 62 °C and 65 °C (Table 2). While there was clear amplification in 58 °C and 62 °C, no clear products were observed at 65 °C in EMU04, EMU06, EMU11, EMU14 and EMU15. However, EMU07, EMU09 and EMU10 resulted clear products in all the temperatures in all the samples. Human DNA resulted clear products with most of the tested SSRs with matching size of elephants. Therefore, there is less possibility to discriminate human DNA contaminations either in gel electrophoresis or by capillary electrophoresis.

**Table 2:** Number of bands detected in primers with the variation of annealing temperature and DNA type.

Primer	T_a_ (°C)														
	Elephant dung DNA sample 09			Elephant dung DNA sample 37			Elephant dung DNA sample 81			Plant DNA mixture			Human DNA		
	58	62	65	58	62	65	58	62	65	58	62	65	58	62	65
EMU03	1	1	1	0	1	0	1	1	0	0	0	0	3	1	0
EMU04	1	1	0	1	1	0	1	1	0	1	1	0	0	1	0
EMU06	1	0	0	1	0	0	1	0	0	0	0	0	0	0	0
EMU07	2	2	2	2	2	3	1	5	4	2	3	3	3	2	3
EMU09	1	1	1	1	1	1	1	1	1	1	1	1	1	1	1
EMU10	1	1	1	2	2	1	1	2	1	1	1	1	3	2	2
EMU11	2	2	0	2	2	0	2	2	0	2	2	0	2	2	0
EMU12	1	1	0	1	0	0	1	1	0	1	0	0	1	1	1
EMU13	3	1	1	2	1	1	1	0	1	1	1	0	2	1	2
EMU14	1	1	0	1	4	0	1	1	0	1	0	0	0	0	0
EMU15	1	1	0	1	1	0	1	1	0	1	1	0	3	2	0
EMU17	2	2	0	1	1	1	2	3	0	1	1	0	2	2	2

### In silico analysis

Overall, majority of the selected primers amplified single or multiple products from plant DNA and human blood DNA. Therefore, the specificity of primers was further tested against available sequence information in National Center for Biotechnology Information (NCBI). Only the products smaller than 500 bp were considered in the BLAST analysis against selected plant and animal genomes (Supplementary Table S2). Except for EMU06 and EMU07, all the other primers had hits in selected genomic sequence data. Further, except LafMS02, LA2, LA6 and EMU10, all the others had hits with comparable size in the human genome. While Kongrit et al. in 2007^48^ reported amplification of four alleles from Asian elephant blood and dung samples for EMU06, we observed clear products only in three out of six blood samples (Table 2). Further, EMU06 resulted amplification in some dung samples. However, it did not result amplification either from plant or human blood DNA tested in the current study. Nevertheless, EMU07 resulted clear multiple products from elephants’ dung DNA samples, plant DNA mix, and human blood DNA.

## Discussion

SSR technique was first introduced by Michael Litt and Jeffrey A. Luty in 1989^94^. Since then, it is probably the most widely used technique in DNA fingerprinting of both animals and plants^95–98^. Human DNA fingerprinting also relies on 13 Short Tandem Repeat (STR) regions and a universally accepted set of primers^99^.

Fernando et al., in 2001^49^ used five sets of SSRs (EMX-1, EMX-2, EMX-3, EMX-4 and EMX-5) for Asian elephant genetic analysis for the first time. Since 2001, the same set of SSR has been used^77^ for both blood DNA^92^ and dung DNA analysis^77^ and all of them were included in this study. Similarly, all the other primers included in this study have been used more than once. Nevertheless, the first set of SSRs for the elephant genetic analysis was developed before the rapid growth of next-generation sequencing data. In the past, the PCR products were separated in polyacrylamide gels and stained with silver nitrate^46,55^. In the current work we used an advanced technique, PCR followed by capillary electrophoresis using a genetic analyzer, also known as fragment analysis^86^. Even a single base-pair length difference can be distinguished using this technique. The selected SSRs have also previously been used for fragment analysis-based studies.

Nevertheless, here we compared the amplification in blood and dung DNA, which has not been done before in a single study. We first used the ten most polymorphic SSRs from the previous research to analyze blood DNA extracted from six elephants housed at the National Zoological Garden, Sri Lanka. Interestingly, only eight out of ten were polymorphic and the total number of alleles and the number of polymorphic loci were less than those of previous studies. Since lower overall genetic diversity among them could be a reason for the next set of experiments, we selected another population of four elephants, including one elephant with Indian origin. We also expanded the list of SSRs from ten to 24, including all the SSRs repeatedly used in previous studies. Further, we selected dung, as the material of choice since obtaining blood from a large number of wild elephants would be a difficult task for any future study. Interestingly, when the same primers used for the blood were used for the analysis of dung DNA, it resulted multiple amplifications in many cases. Those products did not disappear when the annealing temperature increased, suggesting they are to be specific amplifications. To best of our knowledge, there are no reports on testing the specificity of the selected set of SSRs either in the wet lab or in silico. Therefore, the possibility of miscounting and interpretation in previous studies cannot be excluded. However, a recently developed set of SSRs was analyzed in silico to test the possibility of amplification from human since it is a possible contaminant with highly degraded samples^84^. Gugala and colleagues^84^ used NCBI BLAST to search each sequence of the locus against African Savannah elephant, *L. africana* non-redundant database. Then they used the software in silico PCR on the UCSC Genome Browser to query the primers against the human genome. Interestingly, about 57% of the primers picked from the initial analysis resulted hits in the human genome suggesting those primers would result products from possible human genome contaminants. Further, when they conducted the wet lab analysis with SSRs with no hits in the in silico PCR work, template resulted expected products with human DNA. Our work also showed similar results for some primers, for example, EMU06 and EMU07. The necessity of in silico analysis for testing specificity of SSRs is highlighted for other wild animals, for example, leopard^100^.

Nevertheless, the current study suggests that the analysis should extend beyond the human genome especially when dung DNA is used as starting materials. It is even critical when wild-collected or degraded samples are included in the analysis^101^. Except for rice, *Oryza sativa,* no plant selected in the study has fully sequenced and assembled genomes. Even the *O. rhizomatis* could have different SSR motives than *O. sativa* genomic data in the NCBI. Similarly, the Sri Lankan human DNA randomly selected for the study could have differences compared to the sequences available in the database. Some primers resulted multiple hits in the same organism while the others had hits in several organisms. Here, we considered only a subset of eukaryotic organisms with available sequencing data and majority of the possible contaminants in the wilderness; both flora and fauna are yet to be sequenced. Further, we have not considered any microbial genomes, fungal and algal genomes in the analysis. Therefore, the in silico analysis data presented are not complete and possible cross amplifications could be much higher for the same primers. Further, we only considered the products smaller than 500 bp in size. The presence of larger products as observed in wet-lab experiments is possible and such amplification would reduce the efficiency of PCR amplification reaction. Contaminants can be avoided by taking extra precautionary steps when the blood samples are collected from wild or captive elephants. However, such options are not possible when dung or degraded tissue samples are collected from the wild, especially in forensic cases. Therefore, the specificity of primers is a critical factor deciding the success of traditional SSR based methods adopted for such analysis.

During our sample collection, we noticed that bugs and small wild creatures get onto the dung piles within minutes of defecation. Even though we removed them physically before harvesting the outermost mucus layer, their mucus would also have been mixed with the outer mucus layer collected. Therefore, even though we harvested the outermost mucus layer, contaminations could be possible. Further, there are limited options in the type of sample or availability of samples, especially in forensic analysis. Therefore, having an elephant specific set of SSR is a necessity to continue genetic and forensic work with this technology. Based on our study, no primer set out of 24 tested SSRs could be recommended for future work when the elephant dung is used as the starting material. If blood samples are drawn carefully with no human or other contaminations, those with no multiple hits in the elephant genome, for example, EMU06 and EMU07 could still be used. As such, results of the previous studies done with elephant dung would be questionable with the evidence gathered from current findings. Nevertheless, no one could challenge the past since the revolutionary technologies pawed the path for the success of current studies. However, our results suggest the necessity of revisiting available methods.

Alternatively, more specific methodologies with improved statistical power and high genome representation like ddRAD sequencing^57,102,103^, SNP genotyping^57,104^, genotyping-by-sequencing (GBS)^86,105,106^ may need to be applied. Having good coverage Asian and African elephant genome would speed up such attempts.

## Materials and methods

### Sample collection

Blood DNA samples were collected individually from six Asian elephants in the Dehiwala National Zoological Garden, Sri Lanka (Supplementary Figure S3a–f) to a vial with K_3_EDTA. Name, sex, age, morphological characteristics and appearance of elephants were recorded.

For the optimization of dung DNA extraction, elephant dung samples were collected from four domesticated elephants belonging to the Temple of Tooth Relic, Kandy, Sri Lanka. Three of them have Sri Lankan origin while the other one is a donation from India.

A total of 93 dung samples were collected from different locations of Sri Lanka (Fig. 3). The sample collection was carried out by the Department of Wildlife Conservation, Sri Lanka. All the samples were collected with direct sightings. The samples were collected right after the elephant left the site. The collector observed the elephant from the water holes or tanks and waited till they come to drink water where the pictures were taken and morphological characters, for example, approximate size, sex of the elephant and GPS locations of sample sites were recorded. Each sample was given an ID and collected into a polyethene ziplock bag and the samples were packed in a plastic box (Supplementary Figure S3g–h). The boxes were brought to the lab within 24 h as much as possible. The outer mucus layer was carefully and immediately transferred to four 2.0 mL tubes for separate extractions. Another sample from the outer mucus layer and inner bolus were collected to separate 50 mL tubes and stored in − 80 °C for future studies (Supplementary Figure S3i–j). Fibres inside the dung bolus were taken separately and washed thoroughly to remove other impurities. These clean fibres were taken as a source of plant DNA.

Plant samples for DNA extraction were collected from different locations of the country, *Cinnamomum zeylanicum* from Cinnamon Research Station, Palolpitiya, Matara, *Oryza rhizomatis* from Yala National Park, *Santalum album* from Livestock Farm, Udaperadeniya, Peradeniya, and *Strobilanthes zeylanica* from Sripada Peak Wilderness sanctuary. Leaf samples collected were ground with liquid nitrogen and stored in the − 80 °C freezer.

Human buccal cells were obtained from a volunteer individual, after getting a written informed consent clearing the ethical clearance guidelines.

### Optimization of DNA extraction protocol

Elephant blood DNA was extracted using Wizard Genomic DNA Purification kit (Promega, A1120) following the manufacturer’s protocol.

All previous studies depended on fresh dung samples collected less than 24 h time. We also tried to use fresh samples as much as possible. However, since some collection sites were in deep forests/reserves in Sri Lanka, taking them to the lab on time would not possible. Therefore, we optimized a DNA extraction protocol, starting with QIAGEN QIAamp Fast DNA Stool Mini Kit (Cat. No: 51604) which can even be used for samples reach the lab within a week time. We changed the incubation time and temperature combination to reduce PCR inhibitors and get quality DNA. About 100 mg of the dung samples collected were incubated with 1 mL of inhibitex buffer at different temperatures and time intervals; 56 °C overnight incubation, 56 °C 4 h incubation, 56 °C 3 h incubation, 56 °C 2 h incubation, 70 °C > 1 h incubation and 70 °C 1 h incubation. The rest of the steps followed the manufacturer's guidelines. The extracted DNA was dissolved in 50 µL of Tris–acetate-EDTA (TAE) buffer provided with the kit. DNA quality and integrity were tested by running samples on 1% Agarose gel and using Nanodrop 2000 Spectrophotometer (Thermo Scientific).

### Plant and human DNA extraction

Plant DNA was extracted using QIAGEN DNeasy Plant Mini Kit (Cat. No. 69104) following manufacturers’ guidelines and stored in Tris–EDTA (TE) buffer supplied in the kit, at − 20 °C. About 1 µg of DNA from each species was mixed to obtain the plant DNA mix. DNA from the fibres collected from the dung bolus was also extracted using the same kit. Human DNA was extracted using the method described by^107^ and dissolved in TE buffer (Sigma, Cat no: 93283).

### Selection of SSRs

A total of 24 SSRs were selected based on previous literature. Of those, 17 have resulted polymorphic loci in Asian elephants where seven were used for African elephants (Supplementary Table S1). All the primers were synthesized at the Integrated DNA technologies IDT, Singapore and M13 forward sequence (5′-TGTAAAACGACGGCCAGT-3′) was added to the 5′ end of one set of forward primers to facilitate fluorescent dye labelling in PCR amplicon to be used for capillary sequencing. Therefore, for each SSR, two sets of forward primers were available with one set of reverse primer.

### Optimization of PCR with blood DNA

For initial testing, the most polymorphic SSRs were selected. As such, PCR was carried out with ten SSRs, EMU04, EMU06, EMU07, EMU09, EMU10, EMU11, EMU12, EMU13, EMU15 and EMU17 by varying the reaction conditions and thermocycler conditions—‘Touchdown 55’ (Supplementary Table S3). PCR amplicons were examined on 1.5% agarose gel with 0.5 µL of SYBR Safe (Applied Biosystems) under UV light using the Imager—ChemiDoc BioRad (Software version 6.0.1.34) using the standard filter settings. Amplified products were subjected to capillary electrophoresis in an ABI PRISM 3100 Genetic Analyzer (Macrogen, Korea).

### Identification of polymorphic SSRs for dung DNA analysis

DNA extracted from the dung samples collected from four elephants rearing at the Temple of the Tooth Relic, Kandy were PCR amplified under the optimized PCR conditions ‘Touchdown 55’ with the selected 24 SSRs. The PCR products were visualized on 1.5% Agarose gels with SYBR safe dye under UV light and subjected to capillary electrophoresis using the ABI PRISM 3100 Genetic Analyzer (Macrogen, Korea).

### Analysis of wild samples

LafMS02 was the most polymorphic locus and was selected for amplification of 93 wild elephant samples under the same experimental setup.

### PCR with plant and blood DNA

Twenty four selected SSRs specifically designed for elephant fingerprinting work, were tested with the plant DNA mixture. Blood and dung DNA from the same elephant (sample no: 66) and DNA extracted from fibers inside the dung bolus were also included in the analysis. The same plant DNA was reamplified with EMU07, EMU09, EMU10, EMU13, EMU14, EMU15, LafMS06, EMX-4 and LA06 to check the repeatability. The same PCR reaction mixture and the thermocycler conditions ‘Touchdown 55’ were used. (Supplementary Table S3). The products were resolved in either in 1.5% or 2% agarose gels.

### Analysis with high annealing temperature

Previous studies suggested annealing temperature of 58 °C for most of the primers. Three different touchdown annealing temperatures were tested to get rid of unspecific amplification; ‘Touchdown 58’ ‘Touchdown 62’ and ‘Touchdown 65’ (Supplementary Table S3). Three randomly selected dung DNA samples, the same plant DNA mixture mentioned above and a human blood DNA sample were included in the analysis. The effect of FAM labelling was tested for primers with and without labelling.

### In silico analysis

The specificity of each primer set was assessed on August 15th, 2019 using NCBI primer BLAST (https://www.ncbi.nlm.nih.gov/tools/primer-blast/) (non-redundant database) against the same set of organisms selected from the dropdown menu options: the BLAST parameters included, maximum target size, 500 bp minimum of blast target sequence = 10,000, Blast expected value = 10 and maximum pair to screen = 2000.

### Data analysis

Chromatograms were visualized using Peak Scanner Software Version:1.0 (Applied Biosystems). GS350 and GS500 size standards were used in capillary electrophoresis analysis. The scale of Y axis and X axis were zoomed according to the length size of the product. Well distinctive peaks in the chromatogram were scored as discrete variables as presence (1) and absence (0). In order to calculate the polymorphic percentage, total number of loci and polymorphic loci were counted. For the phylogenetic analysis, binary data matrix was bootstrapped for 1000 resamples using *seqboot* program in PHYLIP-3.698 phylogenetic inference package^108^ and subjected to *restdist* program to generate distance matrix^109^. Then neighbor joining program was used to construct a tree by Unweighted Pair Group Method with Arithmetic means (UPGMA) method of clustering and the tree generated was viewed with FigTree 1.4.2^110^.

### Ethical approval

The study was conducted under the research permit number: WL/3/2/2017/1 provided by the Research Committee of the Department of Wildlife Conservation, Sri Lanka. All the methods were carried out in accordance with relevant guidelines and regulations for animals. Human genomic DNA was obtained from the buccal cells collected and stored in Department of Biochemistry and Molecular Biology, Faculty of Medicine, University of Colombo, Sri Lanka. Ethical approval was obtained from the ethics review committee, Faculty of Medicine, University of Colombo, Sri Lanka and it was approved for humans (Ethical approval No: EC-12-138). Buccal cells were collected after getting the informed consent from all the subjects. All the methods were carried out in accordance with relevant guidelines and regulations.

## Supplementary Information

Supplementary Information.
