# Supplementary material for: Revisiting traditional SSR based methodologies available for elephant genetic studies
Source: Sci Rep. 2021 Apr 22;11:8718. doi: 10.1038/s41598-021-88034-9 (PMC8062488; doi:10.1038/s41598-021-88034-9)

Revisiting traditional SSR based methodologies available for elephant genetic studies

Marasinghe, M.S.L.R.P.[a1], Nilanthi, R.M.R.[a1], Hathurusinghe, H.A.B.M.[b1], Sooriyabandara, M.G.C. [a1], Chandrasekara, C.H.W.M.R.B.[b1], Jayawardana, K.A.N.C. [a], Kodagoda, M.M. [b], Rajapakse, R.C. [c], Bandaranayake, P.C.G.[b*]

[a] *Department of Wildlife Conservation, 811/A, Jayanthipura Road, Battaramulla, 10120, Sri Lanka.*

[b] *Agricultural Biotechnology Centre, Faculty of Agriculture, University of Peradeniya, Peradeniya, 20400, Sri Lanka*

[c] *Department of National Zoological Gardens, Anagarika Dharmapala Mawatha, Dehiwala, 10350, Sri Lanka.*

[1]These authors contributed equally

***Corresponding author**

pradeepag@agri.pdn.ac.lk

pgunathilake@ucdavis.edu

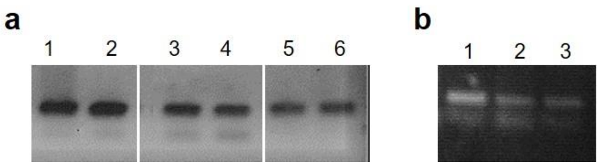

Supplementary Figure S1: Optimization of elephant dung DNA extraction

a. DNA extraction with different incubation times and temperatures using QIAGEN QIAamp Fast DNA Stool Mini Kit (Cat. No: 51604). Subsamples were lysed using 1 mL of lysis buffer provided by the kit for different temperatures for different times while shaking at 700 rpm. The rest of the steps followed the instructions provided by the kit. The extracted DNA was amplified with EMU15 from dung DNA using 'touchdown 55' algorithm. (1). 56 ^{o}C Overnight incubation; (2). 56 ^{o}C 4h incubation; (3). 56 ^{o}C 3h incubation; (4). 56 ^{o}C 2h incubation; (5). 70 ^{o}C >1h incubation; (6). 70 ^{o}C 1h incubation

b. Effect of storage condition of dung samples. The subsamples were stored at 4 ^{o}C for 4 weeks and DNA was extracted using the QIAGEN QIAamp Fast DNA Stool Mini kit (Cat.No: 51604) during the periods of 2 weeks and 4 weeks. The DNA was quantified using NanoDrop Spectrophotometer. In order to check the quality of DNA for PCR, the extracted DNA were amplified with EMU15 using dung DNA following 'touchdown 55' algorithm. (1) Fresh DNA; (2) Mucus layer stored for 2 weeks; (3) Mucus layer stored for 4 weeks

Full-length gels are presented in Supplementary Figure S8.

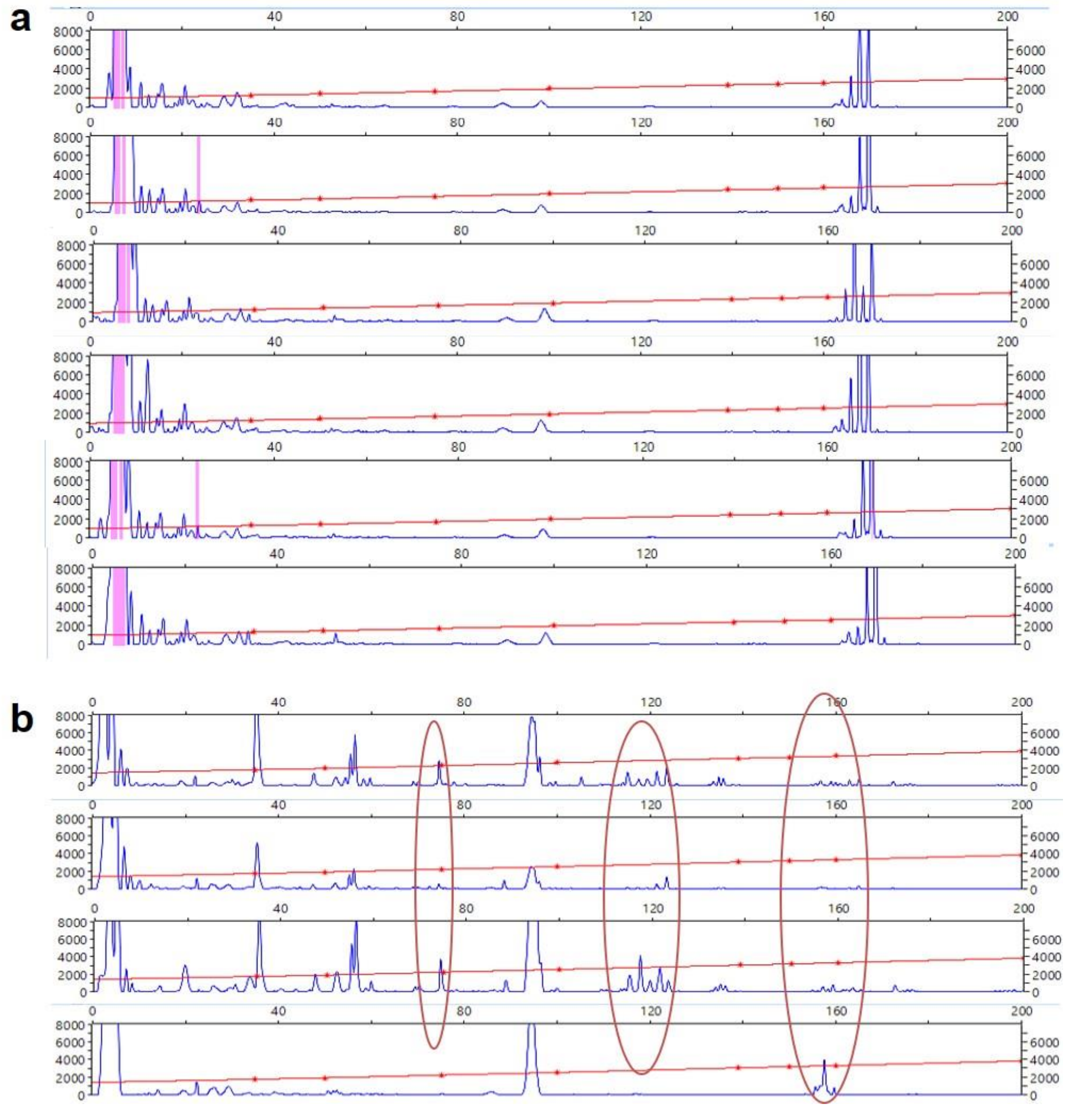

Supplementary Figure S2: Representative capillary Electrophoresis electropherogram

The PCR products with FAM M13 labelled were subjected to capillary electrophoresis using the ABI PRISM 3100 Genetic Analyzer and Electropherograms were visualized using Peak Scanner Software Version 1.0 (Applied Biosystems). The Analysis method - Sizing default; Size standards- GS350 and G500. The X axis of the plots were scaled according to the length size of the product. Only the peaks less than 500 bp height were counted. The peaks were counted considering the peak height which are lesser than 500. **(a).** Electropherogram for blood DNA with primer EMU04 **(b).** Electropherogram for dung DNA with primer EMU04. The unspecific peaks appeared in dung DNA other than in blood DNA are circled in red.

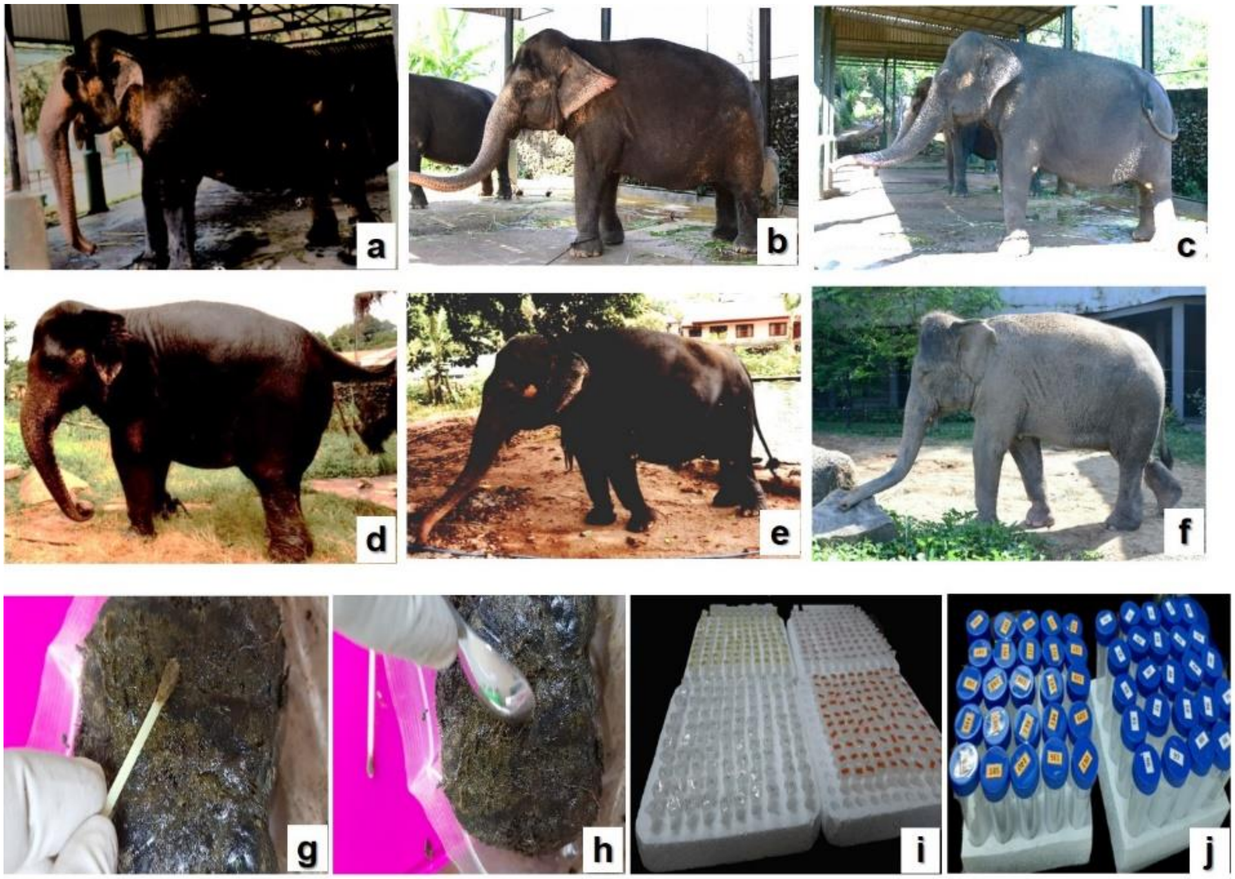

Supplementary Figure S3: Elephant phenotypes and sample collection procedure

a-f. Asian elephants in Dehiwala Zoological Garden, Sri Lanka. 10 mL of blood was collected from the elephants, into tubes containing 50 µL of 50 mM EDTA to prevent coagulation. The tubes were inverted multiple times after blood is added. **a-** Bandula; **b-** Namalee; **c-** Devi; **d-** Ganga; **e-** Indi; **f-** Khema

g-j. Sample collection and storage. **g-h;** Separation of the outermost mucous layer using cotton swab and spatula- The outermost mucous layer of each dung sample was safely skimmed off using a cotton swab and dissolved in the lysis buffer. The layer removed from the spatula were separately stored for long term storage. **i-j;** Storage of samples in 4 ºC and -80 ºC for short term and long term.

Supplementary Table S1 Characteristics of 24 microsatellite markers selected.

No	Locus	Forward Primer Sequence (5'-3')	Reverse Primer Sequence (5'-3')	T_a (ºC)	References
	M13 FAM	6-FAM-TGTAAAACGACGGCCAGT			
1	LafMS02	GAAACCACAACTTGAAGGG	TCGCTTGTAAGAAGGCGTG	62	[54]
2	LafMS06	AGCTGTCCTAAGTCATAAATACACA	ACAGCCACTGAAACCCCATG	58	[52]
3	EMX-1	AGGACTTATTTGCTTAGATGG	AGGCAATGTTTCGTTCTGT	64	
4	EMX-2	CCCATGAGTCGGAATCCACTT	CCATAGGGTTGCCAAGGAATG	70	
5	EMX-3	CATGGTTAACTCATTGCTTGC	GTGTTCCCTCCCTCTCATCAT	64	[52]
6	EMX-4	AGTTCGTGTCTCGGTGCTGTA	GTATGCTGATGGAAATGTCTA	61	
7	EMX-5	AAATAGGAAAAGTCTGAGGTT	CCCCTGGATTTTCTTCACCTG	59	
8	LA2	CTTGGTGGGAGTCATGACCT	GGAGAAATGACTGCCCGATA	58	
9	LA3	TACTCTGCTCCTCTGCCTATCC	GCAGAATTTTGGTCTTGGAGG	55	
10	LA4	GCTACAGAGGACATTACCCAGC	TTTCCTCAGGGATTGGGAG	54	[49]
11	LA5	GGGCAGCCTCCTTGTTTT	CTGCTTCTTTCATGCCAATG	52	
12	LA6	AAAATTGACCCAACGGCTC	TCACGTAACCACTGCGCTAC	57	
13	EMU03	AGAAGCAAAACCCATGAAGC	TTGAAACTTGCCAGCCTCTT	58	
14	EMU04	TGACTCTCCCTCTTCTGCATC	GGCTGAGAGGGAAAGAAATTG	58	
15	EMU06	TTTTTGGGGCTAGAAACTGG	CCCAGTGTTCAATAGATGCTTT	58	
16	EMU07	GAGCAGTGCCTTTCGTGAC	AGCCTGGGAGGTAAGTAGCA	58	
17	EMU09	TCCGTAATTGCACACTTTTAGC	ATGAGGGGTAATGAGGGTCA	58	
18	EMU10	AATCGACTCAGCAGCAACAG	CCAGTAAATCCATATCACTCGTC	58	
19	EMU11	CAATATGGGTGTGGGTTTCC	GAAATGCAGCATAAATAATATCA TGG	58	[48]
20	EMU12	CCAAAGAAGACCCATGTTCC	CTGACTATGGGGGAGACTGC	58	
21	EMU13	GTATTTGGGCTGGCATGGT	GTGGGGTCTGTGGTCAAGTG	58	
22	EMU14	GCCTACATGCAGGGTTTGC	TGAGCCTCTGGCATTTATGA	58	
23	EMU15	TTCGGGATGTTCTCTTCTGT	GGGGCTTAACTAATAGGCTTCA	58	
24	EMU17	CACTCAGAGTTCCAAGAAGCAG	TGCCAGCCATTTCCTCTC	58	

Supplementary Table S2: Summary of *in-silico* PCR against NCBI Primer-BLAST database for 24 primers.

Selected 24 primers from the literature, forward and reverse primer sequence and product length according to the literature are stated below in the table. In order to evaluate the specificity, each primer set was tested using NCBI Primer-BLAST non-redundant database against the available organisms selected from the dropdown menu option. The organism which had blasts smaller than 500 bp product size was taken to the analysis. The organism which had hits, their product size, and primer orientation are mentioned.

Primer	Forward Primer Sequence (5'-3')	Reverse Primer Sequence (5'-3')	Reference	Loci size according to lit.	Blood/ dung	Product length according to BLAST analysis	Organisms which blast with the primers	Product Size	Primer Orientation
LafMS02	GAAACCACAACTTGAAGGG	TCGCTTGTAAGAAGGCGTG	41<br>33<br>54	136-168	Blood And dung	147	*Stomoxys calcitrans* (stable fly/ dung fly)	279	(F/R)
							*Ipomoea triloba (*flowering plant)	104	(R/R)
							Dimocarpus longan (fruit)	39	(F/F)
							Vitus vinifera contig (grapes)	92	(F/F)
								324	(R/R)
LafMS06	AGCTGTCCTAAGTCATAAATACACA	ACAGCCACTGAAACCCCATG	52	138-156	blood	144	*Homo sapiens*	177	(R/R)
EMX-1	AGGACTTATTTGCTTAGATGG	AGGCAATGTTTCGTTCTGT	49<br>41	137-152	Blood and dung	151	*Oryza sativa*	356	(F/R)
							Oryza sativa	358	(F/R)
							Oryza sativa	107	(F/R)
							Oryza sativa	212	(R/R)
							Spirodela polyrhiza (duckweed)	66	(R/R)
							Homo sapiens	359	(R/R)
EMX-2	CCCATGAGTCGGAATCCACTT	CCATAGGGTTGCCAAGGAATG		217-223	Blood and dung	223	*Homo sapiens*	232	(R/R)
Continued							*Trichechus manatus latrirostis* (sea cow)	214	(F/R)

						Procavia capensis (rabbit)	191	(F/R)
						Procavia capensis (rabbit)	143	(F/R)
						Procavia capensis (rabbit)	436	(F/R)
						Procavia capensis (rabbit)	439	(F/R)
						Procavia capensis (rabbit*)*	426	(F/R)
						Cyprinus carpio carp)	428	(F/F)
.						*Trichechus manatus latrirostis* (sea cow)	199	(F/F)
EMX-3	CATGGTTAACTCATTGCTTGC	GTGTTCCCTCCCTCTCATCAT	238-254	Blood and dung	254	*Homo sapiens*	405	(F/R)
						Oryza sativa	225	(R/R)
						Oryza sativa	266	(R/R)
						Oryza sativa	132	(R/R)
						Oryza sativa	233	(R/R)
						Oryza sativa	241	(R/R)
						Oryza sativa	116	(R/R)
						Oryza sativa	242	(R/R)
						Oryza sativa	236	(R/R)
						Oryza sativa	239	(R/R)
						Oryza sativa	196	(R/R)
						Oryza sativa	230	(R/R)
						Oryza sativa	104	(R/R)
						Oryza sativa	231	(R/R)
						Oryza sativa	266	(R/R)
						Oryza sativa	249	(R/R)
						Oryza sativa	248	(R/R)
						Oryza sativa	232	(R/R)
						Oryza sativa	238	(R/R)
						Oryza sativa	217	(R/R)
Continued						*Oryza sativa*	132	(R/R)
						Oryza sativa	243	(R/R)
						Homo sapiens	101	(R/R)
						Candidatus saccharbacterium	463	(R/R)
EMX-4	AGTTCGTGTCTCGGTGCTGTA	GTATGCTGATGGAAATGTCTA	351-387	Blood and dung	379	*Acromyrmex echinatior (*ant)	388	(F/R)
						Homo sapiens	119	(R/R)
						Homo sapiens	140	(R/R)

						Homo sapiens	235	(R/R)
						Oryza sativa	185	(R/R)
						Salarias fasciatus (marine fish)	351	(R/R)
						Cyprinus carpio (carp)	213	(R/R)
						Cyprinus carpio	414	(F/F)
						Ovis candensis (sheep)	357	(R/R)
						*Lupinus angustifolius (*flowering plant)	310	(F/F)
EMX-5	AAATAGGAAAAGTCTGAGGTT	CCCCTGGATTTTCTTCACCTG	248-263	Blood and dung	256	*Homo sapiens*	36	(F/R)
						Homo sapiens	274	(F/R)
						Ola europaea (olive*)*	277	(F/R)
						Poecilio reticulate (gappi)	229	(F/F)
LA2	CTTGGTGGGAGTCATGACCT	GGAGAAATGACTGCCCGATA	226-241	Blood and dung	227	*Oryza sativa*	250	(F/F)
						Oryza sativa	139	(R/R)
						Oryza sativa	140	(R/R)
LA3	TACTCTGCTCCTCTGCCTATCC	GCAGAATTTTGGTCTTGGAGG	166-172	Blood and dung	169	*Homo sapiens*	118	(F/R)
						Amphiprion ocellaris(clown fish)	352	(F/F)
						Solanum penneili (tomato)	113	(R/R)
LA4	GCTACAGAGGACATTACCCAGC	TTTCCTCAGGGATTGGGAG	111-137	Blood and dung	130	*Homo sapiens*	207	(F/R)
						Homo sapiens	209	(F/R)
						Homo sapiens	212	(F/R)
						Homo sapiens	408	(F/R)
						Homo sapiens	437	(F/R)
						Homo sapiens	467	(F/R)
						Homo sapiens	485	(F/R)
Continued						*Homo sapiens*	282	(R/R)
						Homo sapiens	178	(R/R)
						Homo sapiens	207	(F/F)
						Oryza sativa	391	(R/R)
						Oryza sativa	473	(R/R)
						Oryza sativa	481	(R/R)
						Oryza sativa	433	(R/R)
						Oryza sativa	329	(R/R)
						Oryza sativa	281	(R/R)

LA5	GGGCAGCCTCCTTGTTTT	CTGCTTCTTTCATGCCAATG	130-154	Blood and dung	146	*Homo sapiens*	271	(F/R)
						Homo sapiens	487	(F/R)
						Oryza sativa	269	(F/R)
						Oryza sativa	270	(F/R)
						Cryptomeria japonica (plant)	402	(F/R)
						Cocomyxa subellipsodia (green algea)	270	(F/R)
						Populis euphratica (plant)	446	(F/R)
						Homo sapiens	465	(R/R)
						Homo sapiens	48	(R/R)
						Oryza sativa	120	(F/F)
						Oryza sativa	126	(F/F)
						Oryza sativa	128	(F/F)
						Asterias rubem (star fish)	120	(F/F)
						Medicayo truncatula (flowering plant)	88	(R/R)
.						*Solanum lycapersicum*	436	(R/R)
LA6	AAAATTGACCCAACGGCTC	TCACGTAACCACTGCGCTAC	155-214	Blood and dung	166	*Oryza sativa*	361	(F/R)
						Oryza sativa	409	(F/R)
						Schistasoma mansoni (blood fluke)	123	(R/R)
						Schistasoma mansoni	158	(R/R)
EMU03	AGAAGCAAAACCCATGAAGC	TTGAAACTTGCCAGCCTCTT	137-143	Blood and dung	136	*Homo sapiens*	448	(F/R)
						Rhinatrema bivittatum (worm)	358	(R/R)
						Rhinatrema bivittatum	367	(R/R)
						Rhinatrema bivittatum	423	(R/R)
Continued						*Rhinatrema bivittatum*	355	(R/R)
						Mouse	368	(R/R)
						Homo sapiens	366	(F/F)
						Homo sapiens	355	(R/R)
						Homo sapiens	483	(R/R)
						Cajanus cajan (pigeon pea)	91	(F/F)
						Lotus japonica (legume)	353	(R/R)
						Rhinatrema bivittatum	423	(R/R)
EMU04	TGACTCTCCCTCTTCTGCATC	GGCTGAGAGGGAAAGAAATTG	97-107	Blood and dung	101	*Homo sapiens*	106	(F/R)
						Homo sapiens	481	(F/R)

						Homo sapiens	447	(F/F)
						Homo sapiens	480	(F/F)
						Ipomoea trifida	430	(F/F)
						Arabis alpine (flowering plant)	174	(F/F)
						Vigna angulam (beans)	84	(F/F)
EMU06	TTTTTGGGGCTAGAAACTGG	CCCAGTGTTCAATAGATGCTTT	146-158	Blood and dung	145	-	-	-
EMU07	GAGCAGTGCCTTTCGTGAC	AGCCTGGGAGGTAAGTAGCA	102-122	Blood and dung	114	-	-	-
EMU09	TCCGTAATTGCACACTTTTAGC	ATGAGGGGTAATGAGGGTCA	163-169	Blood and	159	*Culicoides sonorensis* (mosquito)	420	(F/F)
				dung		*Homo sapiens*	327	(R.R)
						Homo sapiens	480	(R/R)
EMU10	AATCGACTCAGCAGCAACAG	CCAGTAAATCCATATCACTCGTC	94-104	Blood and dung	100	*Ovis candensis*	240	(F/F)
						Ovis candensis	127	(F/F)
EMU11	CAATATGGGTGTGGGTTTCC	GAAATGCAGCATAAATAATATCATGG	122-136	Blood and dung	123	*Homo sapiens*	465	(F/F)
EMU12	CCAAAGAAGACCCATGTTCC	CTGACTATGGGGGAGACTGC	120-152	Blood and	147	*Homo sapiens*	82	(F/R)
				dung		*Homo sapiens*	242	(F/R)
.						*Gossypium raimondii* (cotton)	413	(F/F)
EMU13	GTATTTGGGCTGGCATGGT	GTGGGGTCTGTGGTCAAGTG	100-110	Blood and	108	*Homo sapiens*	281	(F/R)
				dung		*Homo sapiens*	343	(F/R)
						Homo sapiens	372	(F/R)
						Oryza sativa	131	(F/R)
						Oryza sativa	179	(F/R)
						Oryza sativa	180	(F/R)
						Oryza sativa	78	(F/F)
Continued						*Homo sapiens*	312	(R/R)
						Homo sapiens	178	(R/R)
						Brassica oleracea (wild cabbage)	105	(R/R)
EMU14	GCCTACATGCAGGGTTTGC	TGAGCCTCTGGCATTTATGA	130-138	Blood and	140	*Homo sapiens*	469	(F/F)
				dung		*Homo sapiens*	436	(R/R)
						Homo sapiens	424	(R/R)
						Homo sapiens	478	(R/R)
EMU15	TTCGGGATGTTCTCTTCTGT	GGGGCTTAACTAATAGGCTTCA	142-154	Blood and	155	*Homo sapiens*	424	(R/R)
				dung		*Homo sapiens*	478	(R/R)
						Homo sapiens	436	(R/R)

						Homo sapiens	119	(F/F)
						Homo sapiens	469	(F/F)
EMU17	CACTCAGAGTTCCAAGAAGCAG	TGCCAGCCATTTCCTCTC		119-137	Blood and dung	122	*Homo sapiens*	69	(F/R)
						Homo sapiens	454	(F/R)
						Homo sapiens	258	(R/R)
						Homo sapiens	197	(R/R)
						Homo sapiens	398	(R/R)
						Homo sapiens	388	(F/F)

Supplementary Table S3: Thermocycler conditions

Touch down PCR carried out with FAM M-13 labelled primer, forward with M13 and conventional reverse primer.

Type of PCR	Thermocycler conditions
'Touch down 55'	Initial Denaturation 95 ^{o}C for 5 min<br>30 cycles of 95 ^{o}C for 1 min, 58 ^{o}C for 30 sec, 72 ^{o}C for 1 min<br>8 cycles of 95 ^{o}C for 1 min, 55 ^{o}C for 30 sec, 72 ^{o}C for 1 min<br>Final Extension 72 ^{o}C for 5 min<br>Hold at 4 ^{o}C
'Touch down 58'	Initial Denaturation 95 ^{o}C for 5 min<br>30 cycles of 95 ^{o}C for 1 min, 62 ^{o}C for 30 sec, 72 ^{o}C for 1 min<br>8 cycles of 95 ^{o}C for 1 min, 58 ^{o}C for 30 sec, 72 ^{o}C for 1 min<br>Final Extension 72 ^{o}C for 5 min<br>Hold at 4 ^{o}C
'Touch down 62'	Initial Denaturation 95 ^{o}C for 5 min<br>30 cycles of 95 ^{o}C for 1 min, 65 ^{o}C for 30 sec, 72 ^{o}C for 1 min<br>8 cycles of 95 ^{o}C for 1 min, 62 ^{o}C for 30 sec, 72 ^{o}C for 1 min<br>Final Extension 72 ^{o}C for 5 min<br>Hold at 4 ^{o}C
'Touch down 65'	Initial Denaturation 95 ^{o}C for 5 min<br>30 cycles of 95 ^{o}C for 1 min, 69 ^{o}C for 30 sec, 72 ^{o}C for 1 min<br>8 cycles of 95 ^{o}C for 1 min, 65 ^{o}C for 30 sec, 72 ^{o}C for 1 min<br>Final Extension 72 ^{o}C for 5 min<br>Hold at 4 ^{o}C

Revisiting traditional SSR based methodologies available for elephant genetic studies

Marasinghe, M.S.L.R.P.[a1], Nilanthi, R.M.R.[a1], Hathurusinghe, H.A.B.M.[b1], Sooriyabandara, M.G.C. [a1], Chandrasekara, C.H.W.M.R.B.[b1], Jayawardana, K.A.N.C. [a], Kodagoda, M.M. [b], Rajapakse, R.C. [c], Bandaranayake, P.C.G.[b*]

[a] *Department of Wildlife Conservation, 811/A, Jayanthipura Road, Battaramulla, Sri Lanka.*

[b] *Agricultural Biotechnology Centre, Faculty of Agriculture, University of Peradeniya, Peradeniya, Sri Lanka*

[c] *Department of National Zoological Gardens, Watthegedara Junction Maharagma, Sri Lanka*

[1]These authors contributed equally

***Corresponding author**

pradeepag@agri.pdn.ac.lk

pgunathilake@ucdavis.edu

<u>**FULL IMAGE GEL PHOTOS WITH DIFFERENT EXPOSURES**</u>

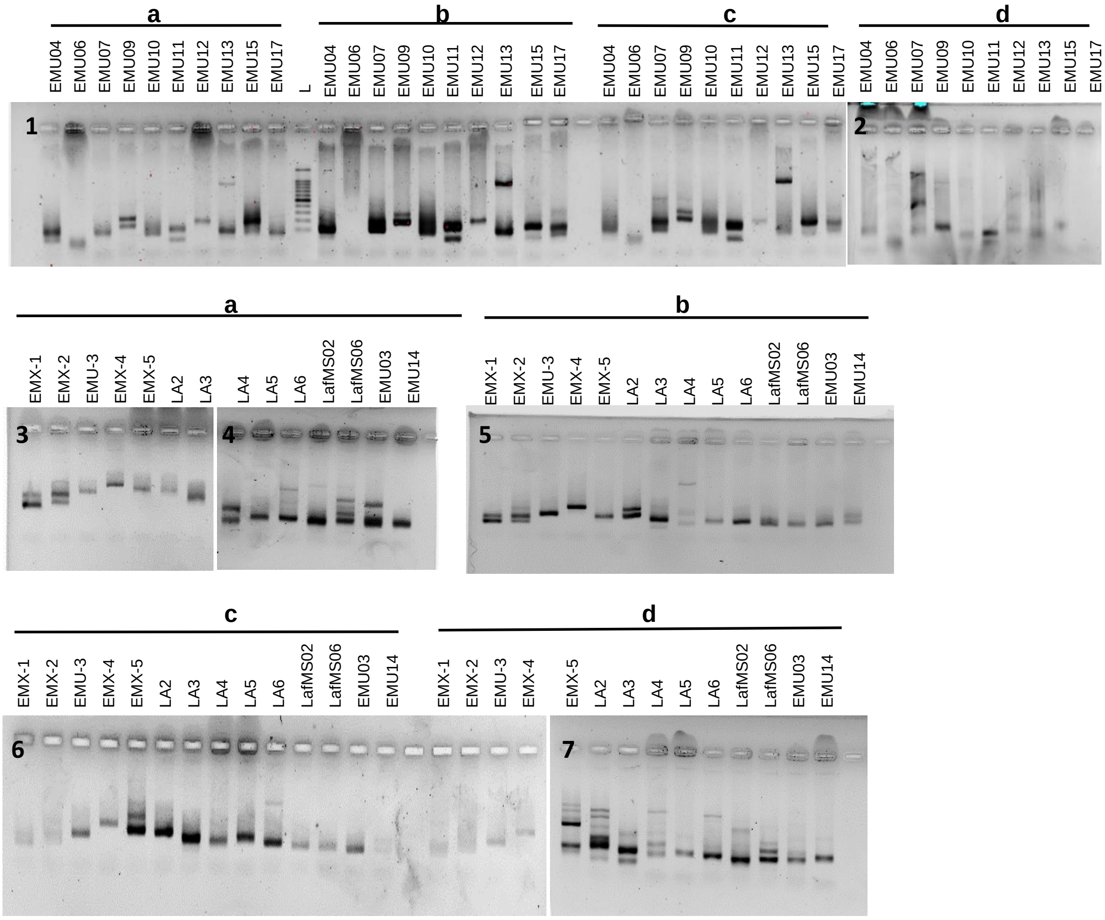

Supplementary full gel image (S4) for figure 2: Identification of polymorphic primers for amplification of dung DNA. Primer names are given on the top of each well. L-100 bp molecular weight marker (Promega, Cat no: G2101), a, b, c – Sri Lankan origin d- Indian origin

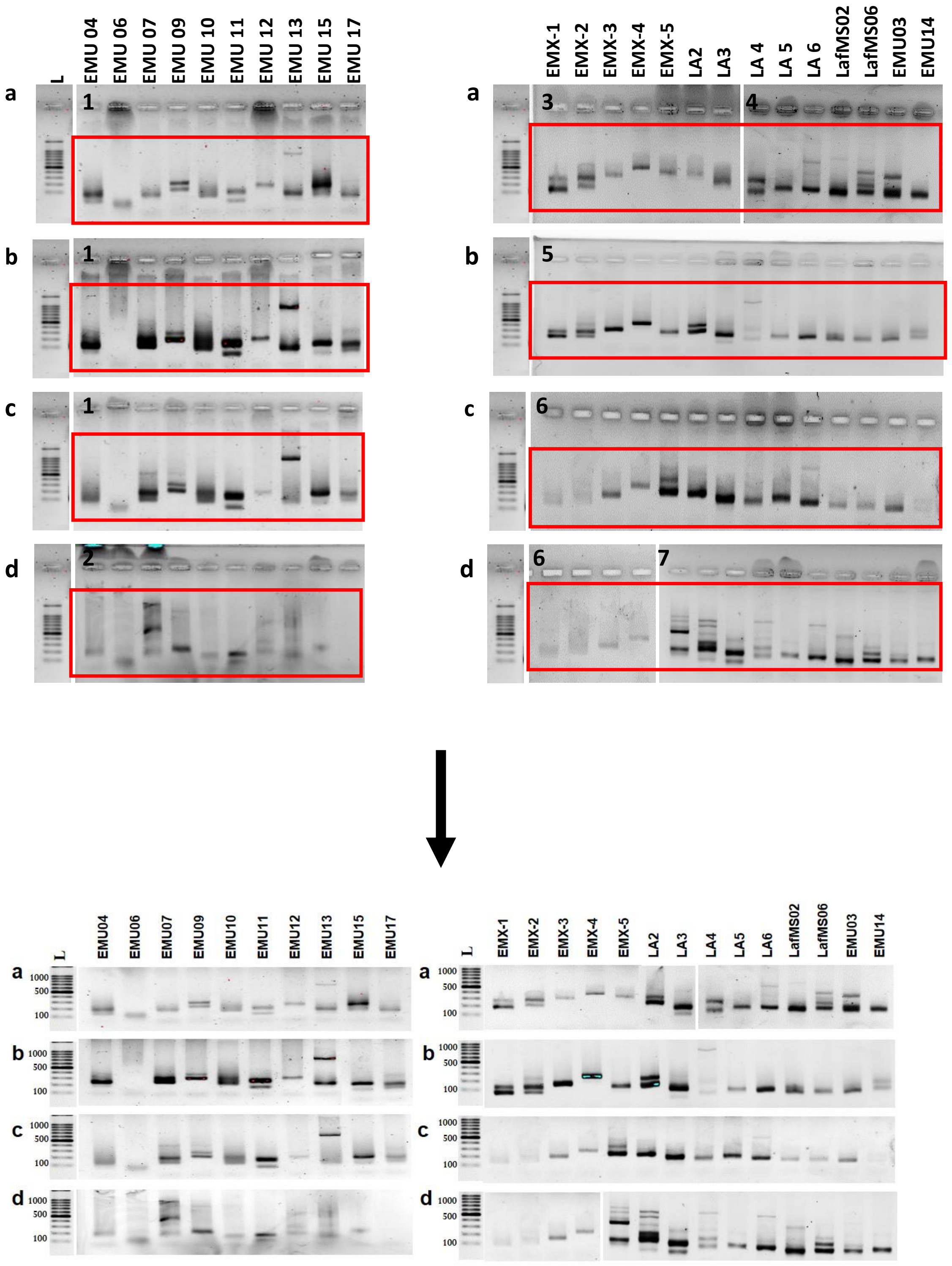

Figure 2: Identification of polymorphic primers for amplification of dung DNA. Primer names are given on the top of each well. L-100 bp ladder (Promega, Cat no: G2101), a, b, c – Sri Lankan origin d- Indian origin.

The above depicted original gel images (**Supplementary full gel image (S4) for figure 2)** were manipulated to create ‘figure 2 in the main article’.

1. Gel image no: 1 in the original article divided in to three parts as elephant a, b and c

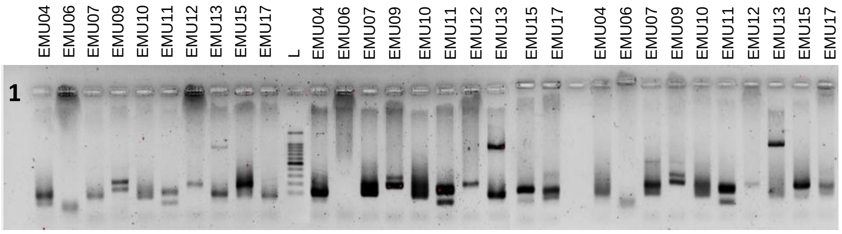

\

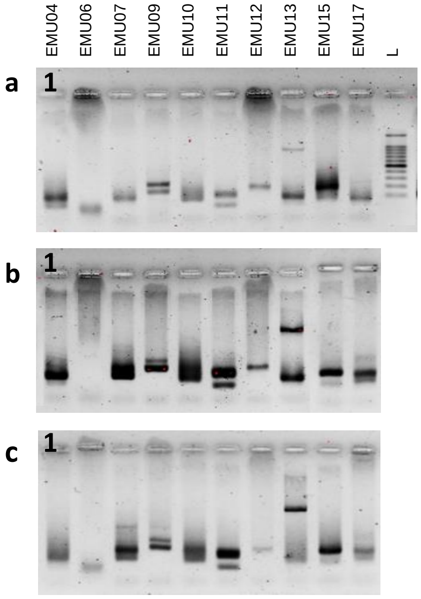

2. Ladder was added to each gel

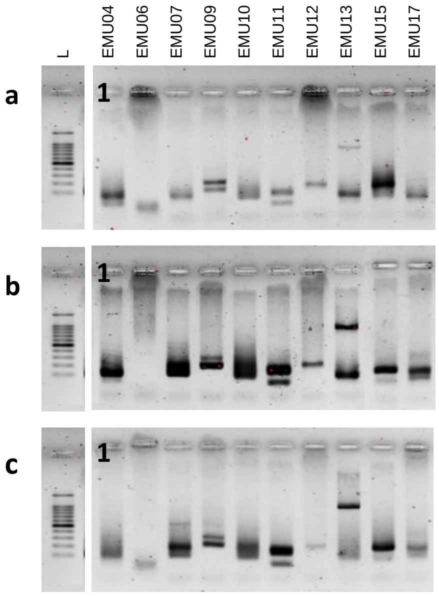

3. Gel image 2 (elephant d) was added separately.

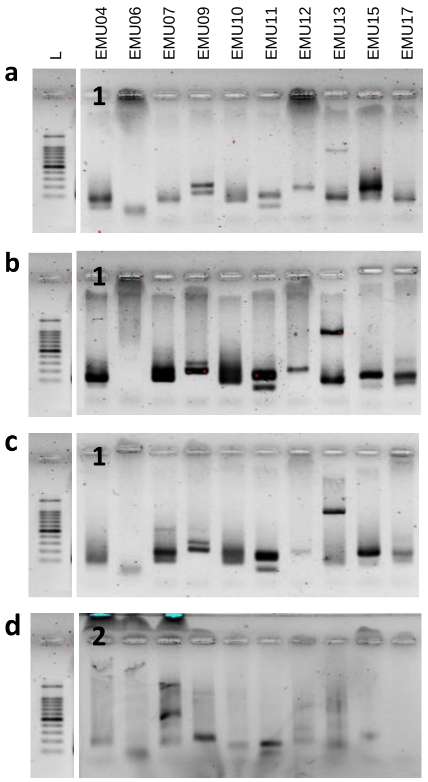

4. Gel images 3 and 4 combined as one image separately to illustrate ‘elephant a’ and same 100 bp ladder was added.

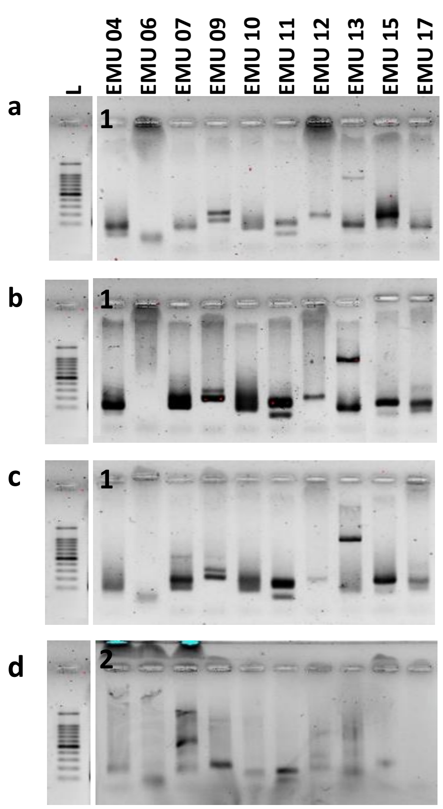

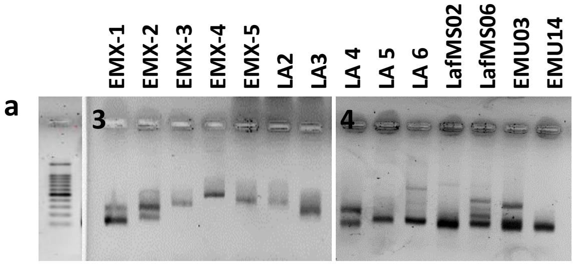

5. Gel image no: 5 separately depicted to illustrate ‘elephant b’

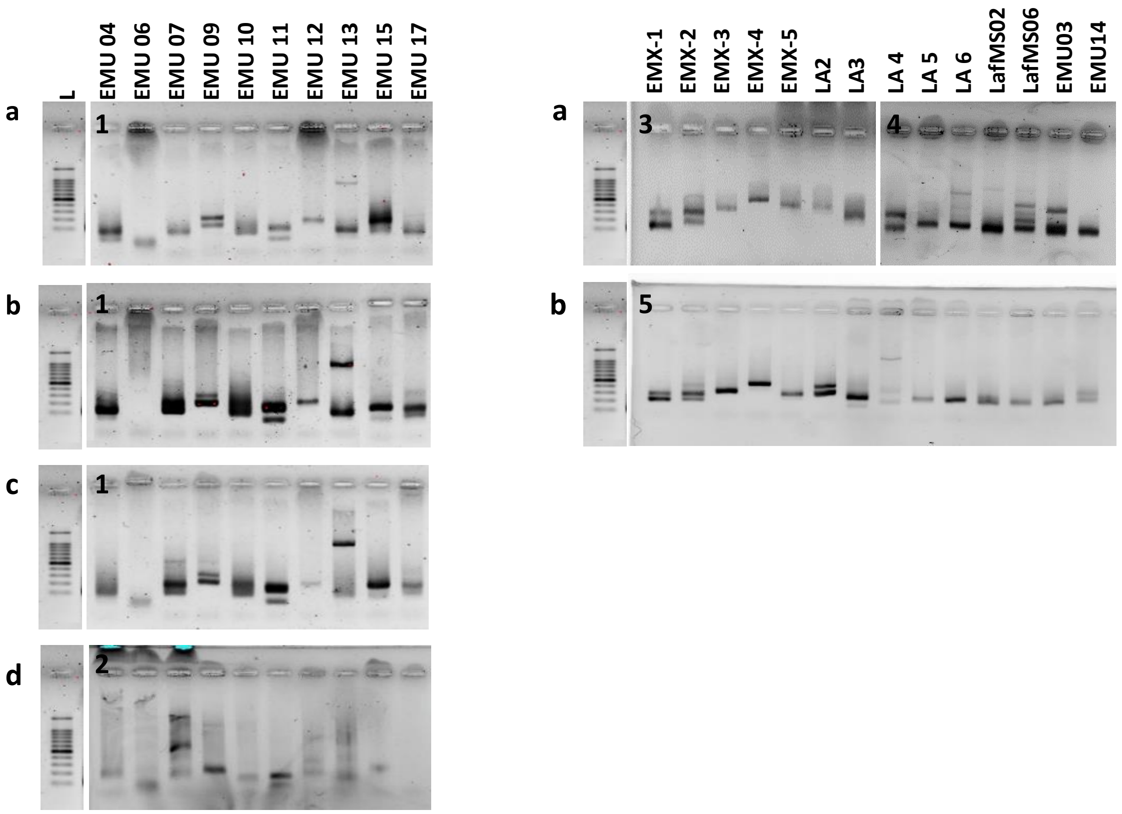

6. Gel image no: 6 illustrates ‘elephant c’. That relevant region is cropped.

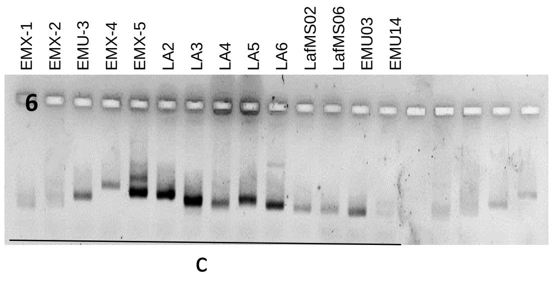

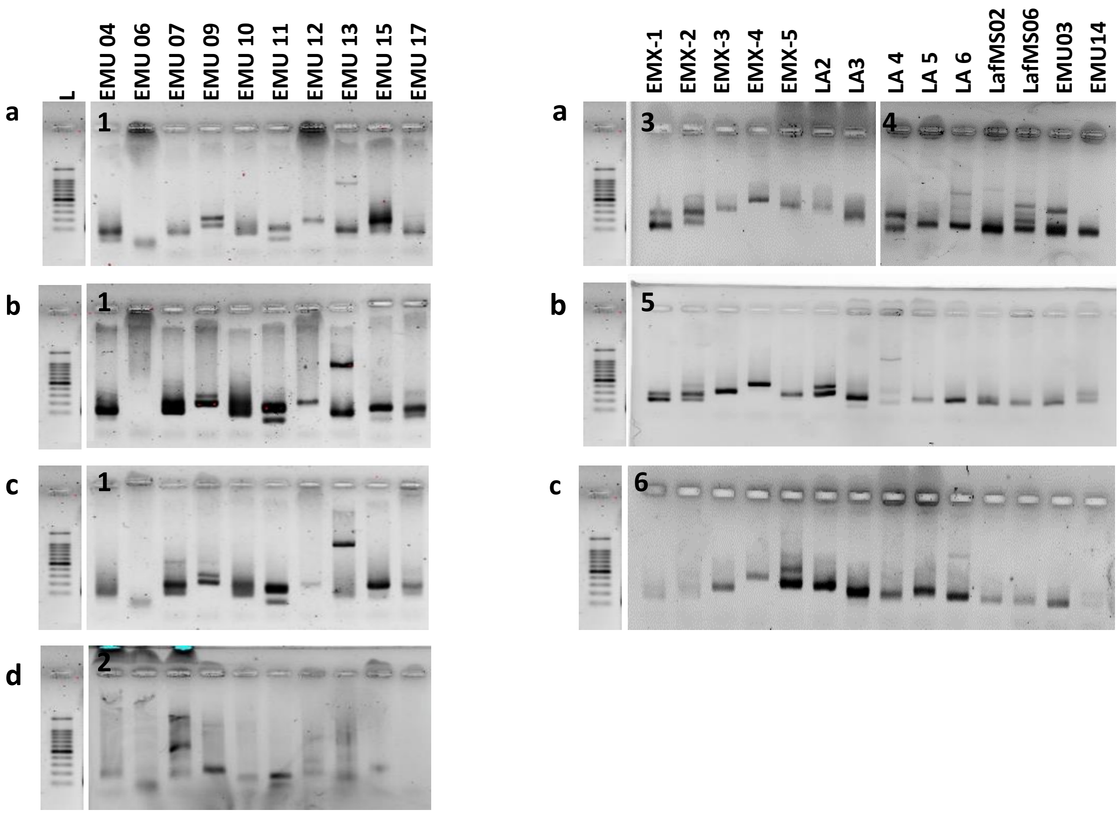

7. To illustrate ‘elephant 6’ the relevant part of gel image no: 6 and gel image no: 7 was combined

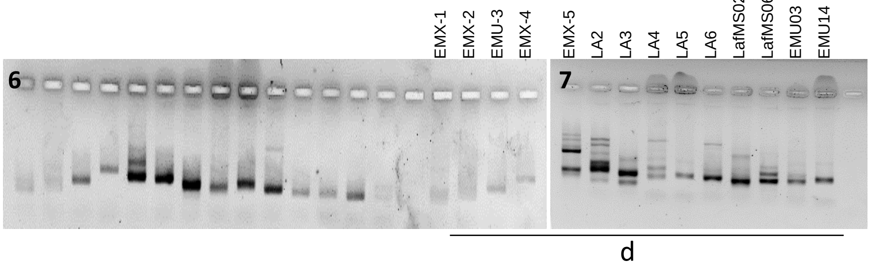

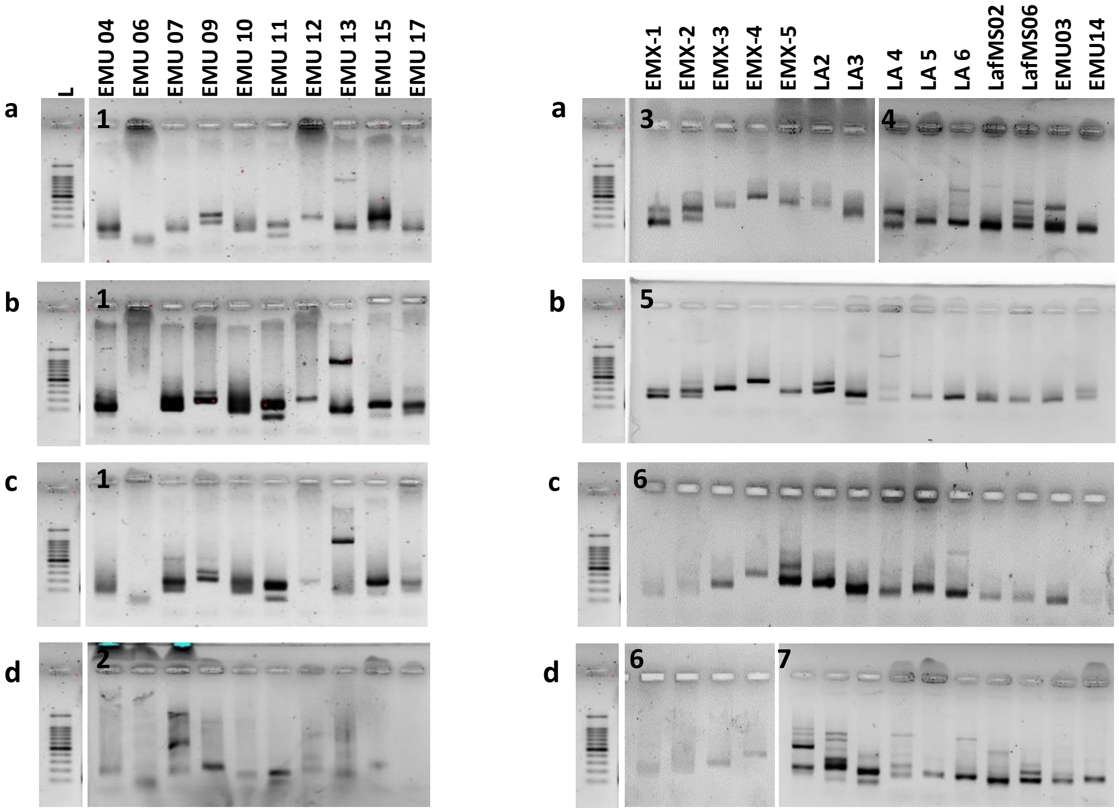

Supplementary full gel image (S4) for figure 2: Identification of polymorphic markers for amplification of dung DNA. Primer names are given on the top of each well. L-100 bp molecular weight marker (Promega, Cat no: G2101), a, b, c – Sri Lankan origin   d- Indian origin

<u>Exposure times-</u>

Gel Number	Exposure time
1	3.109
2	6.285
3	4.425
4	5.598
5	3.013
6	4.813

Gel images with different exposures manually changed.

Exposure 1

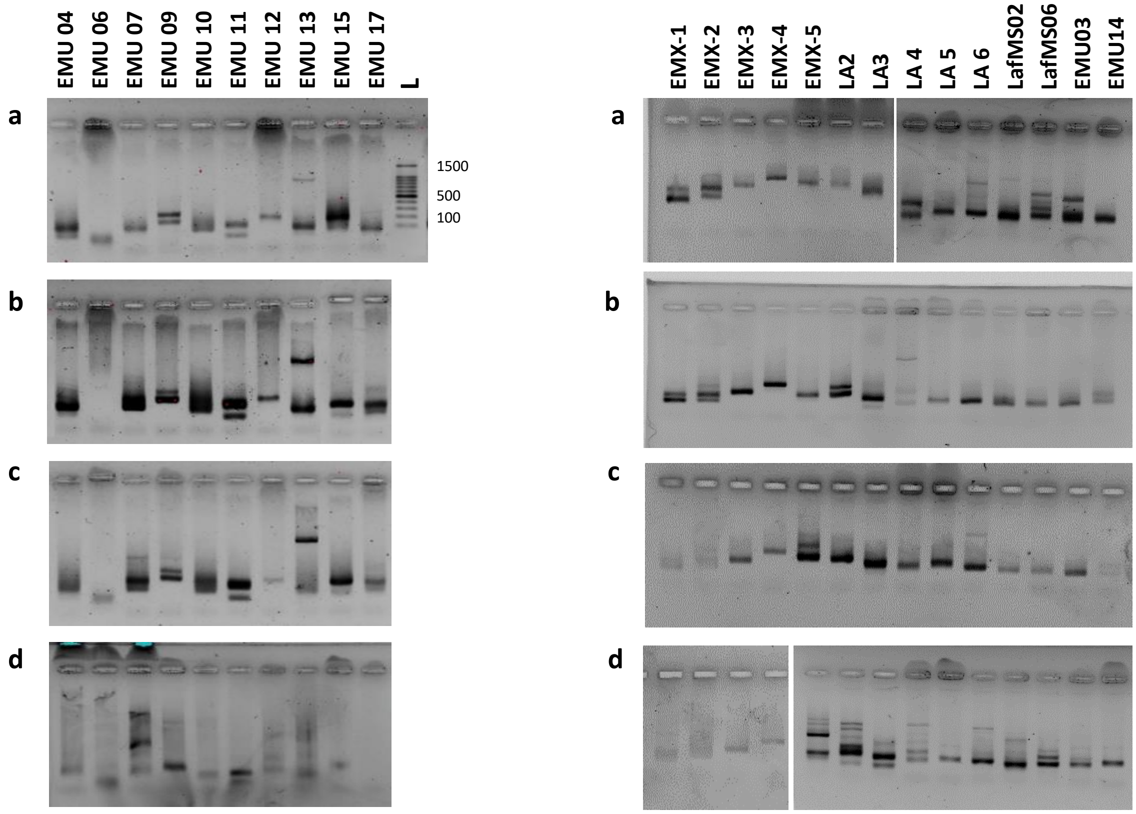

Exposure 2

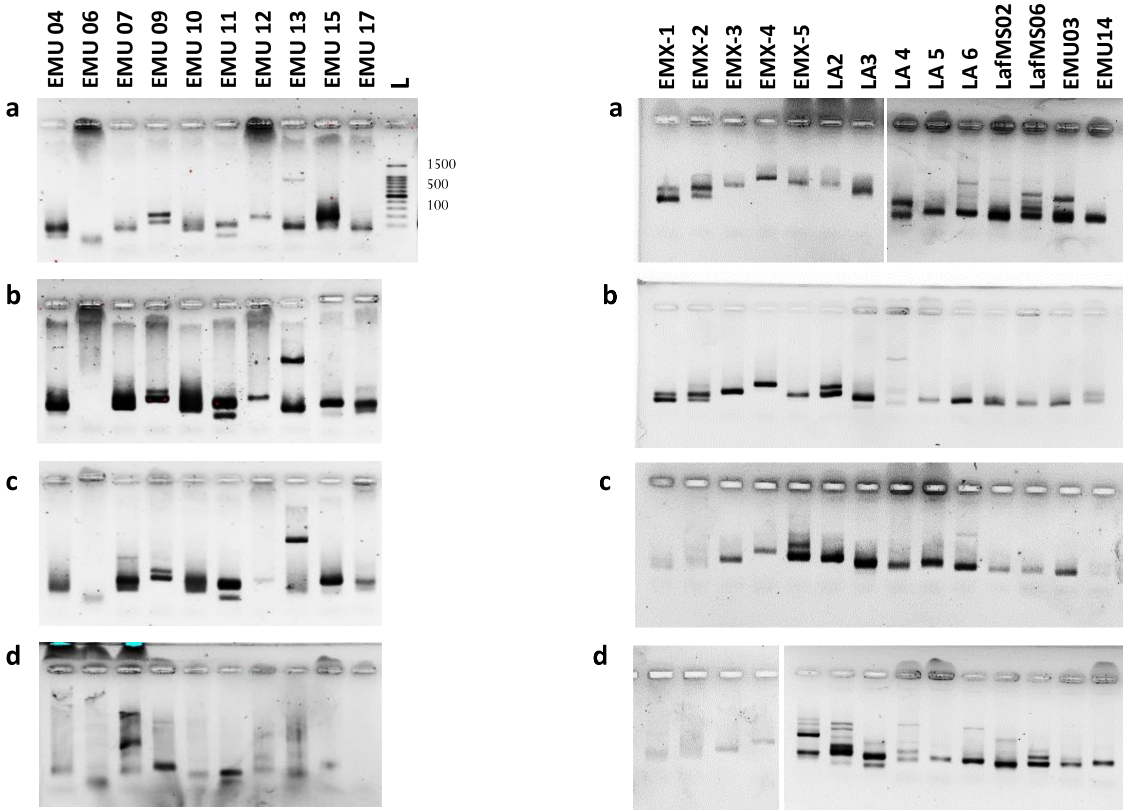
EMU 04
EMU 06
EMU 07
EMU 09
EMU 10
EMU 11
EMU 12
EMU 13
EMU 15
EMU 17
L
1500
500
100
EMX-1
EMX-2
EMX-3
EMX-4
EMX-5
LA2
LA3
LA 4
LA 5
LA 6
LafMS02
LafMS06
EMU03
EMU14
a
b
c
d
a
b
c
d

Exposure 3

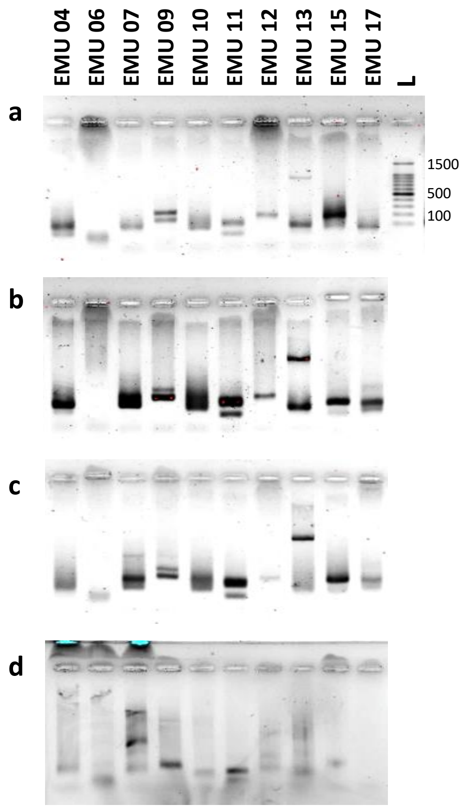
EMU 04
EMU 06
EMU 07
EMU 09
EMU 10
EMU 11
EMU 12
EMU 13
EMU 15
EMU 17
L
a
1500
500
100
b
c
d

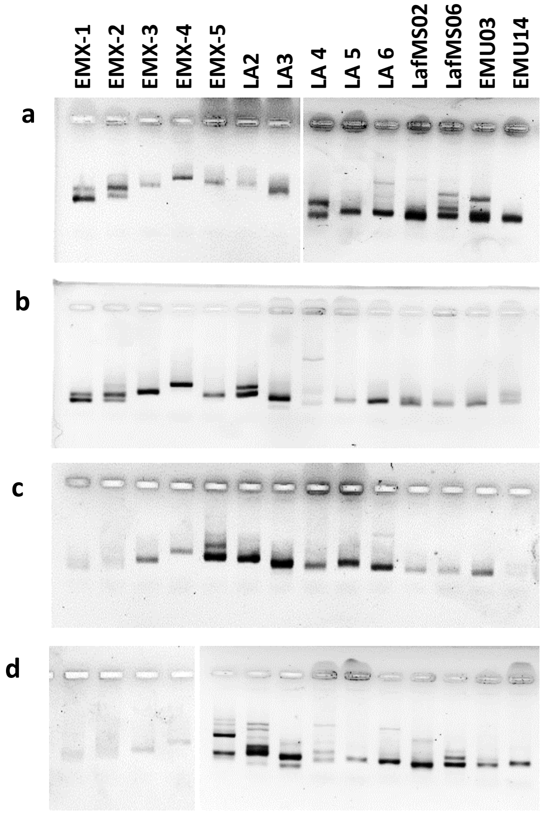
EMX-1
EMX-2
EMX-3
EMX-4
EMX-5
LA2
LA3
LA 4
LA 5
LA 6
LafMS02
LafMS06
EMU03
EMU14
a
b
c
d

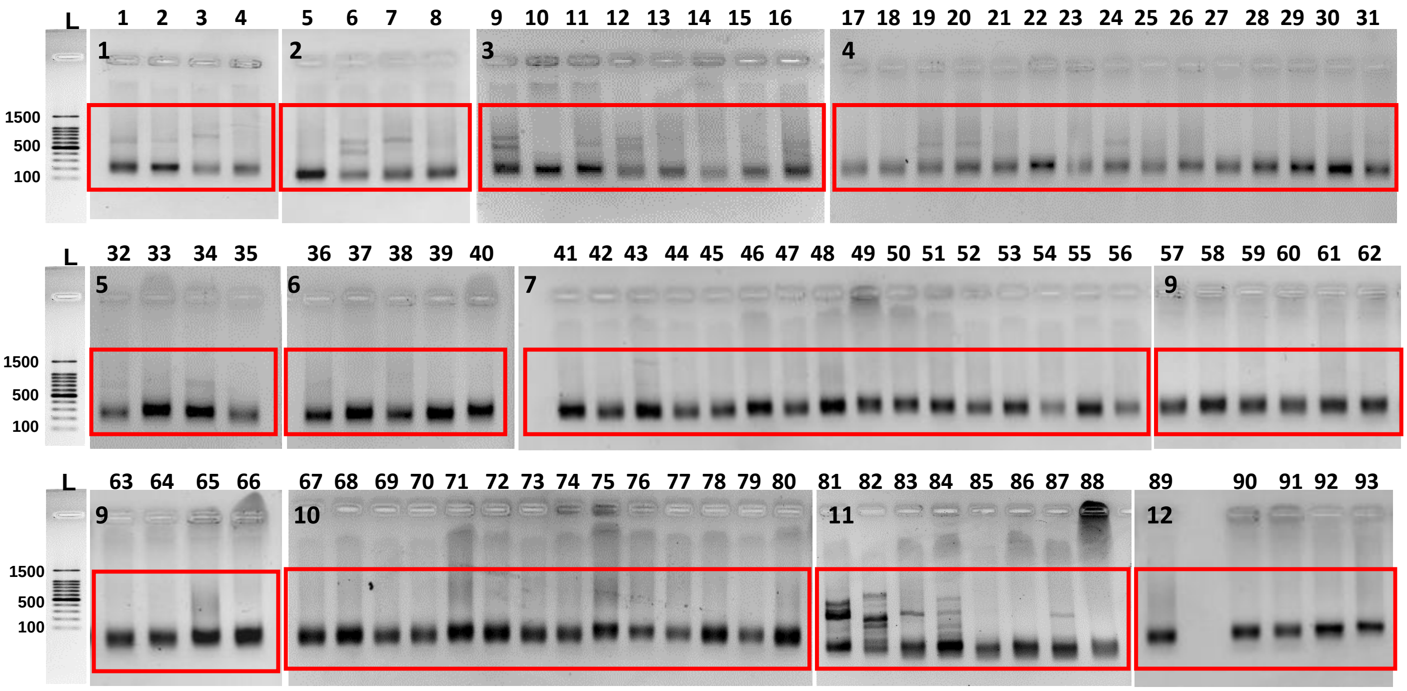

Supplementary full gel image (S5) for Figure 4: Amplification with SSR - LafMS02. L- 100 bp molecular weight marker (Promega, Cat no: G2101), 1- 93 dung DNA samples collected from wild elephants

<u>Exposure times-</u>

Gel Image number	Exposure time
1	3.272
2	3.272
3	4.220
4	4.261
5	4.261
6	4.191
7	4.556
8	4.132
9	3.696
10	4.039

Gel images with different exposures manually changed.

Exposure 1

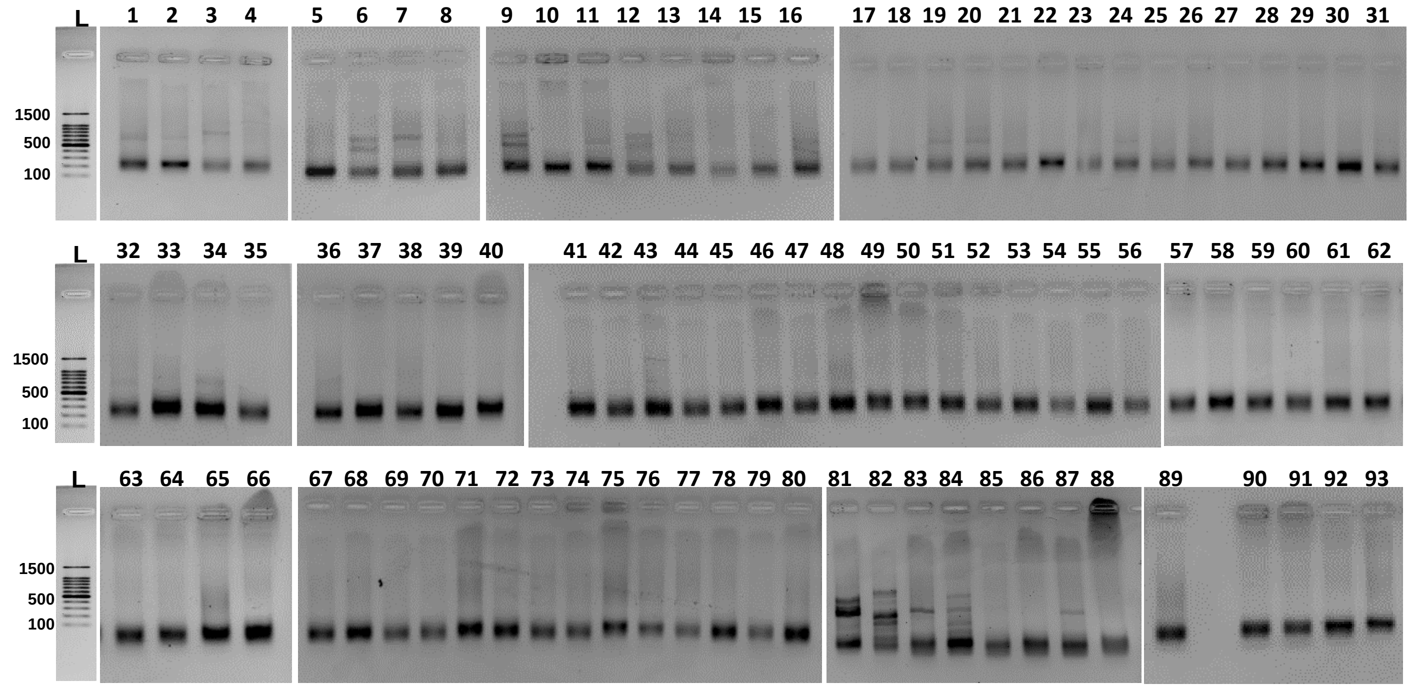

Exposure 2

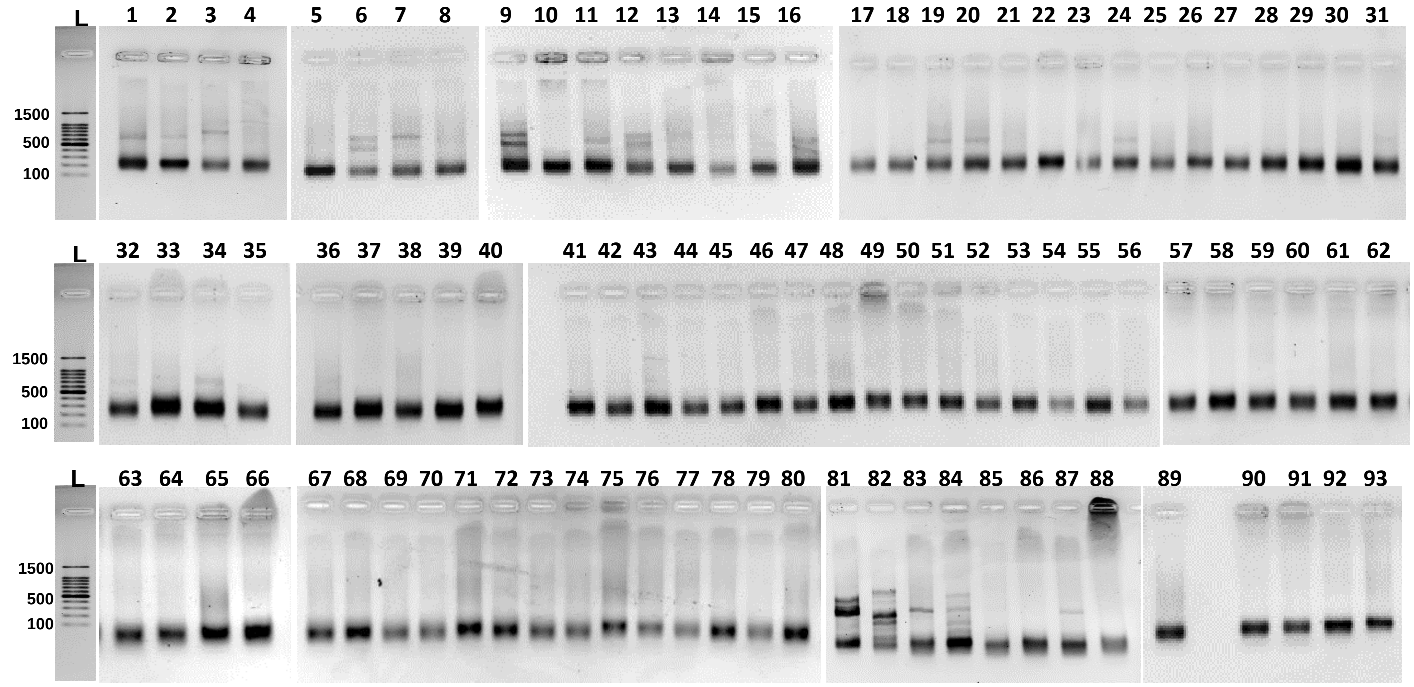

Exposure 3

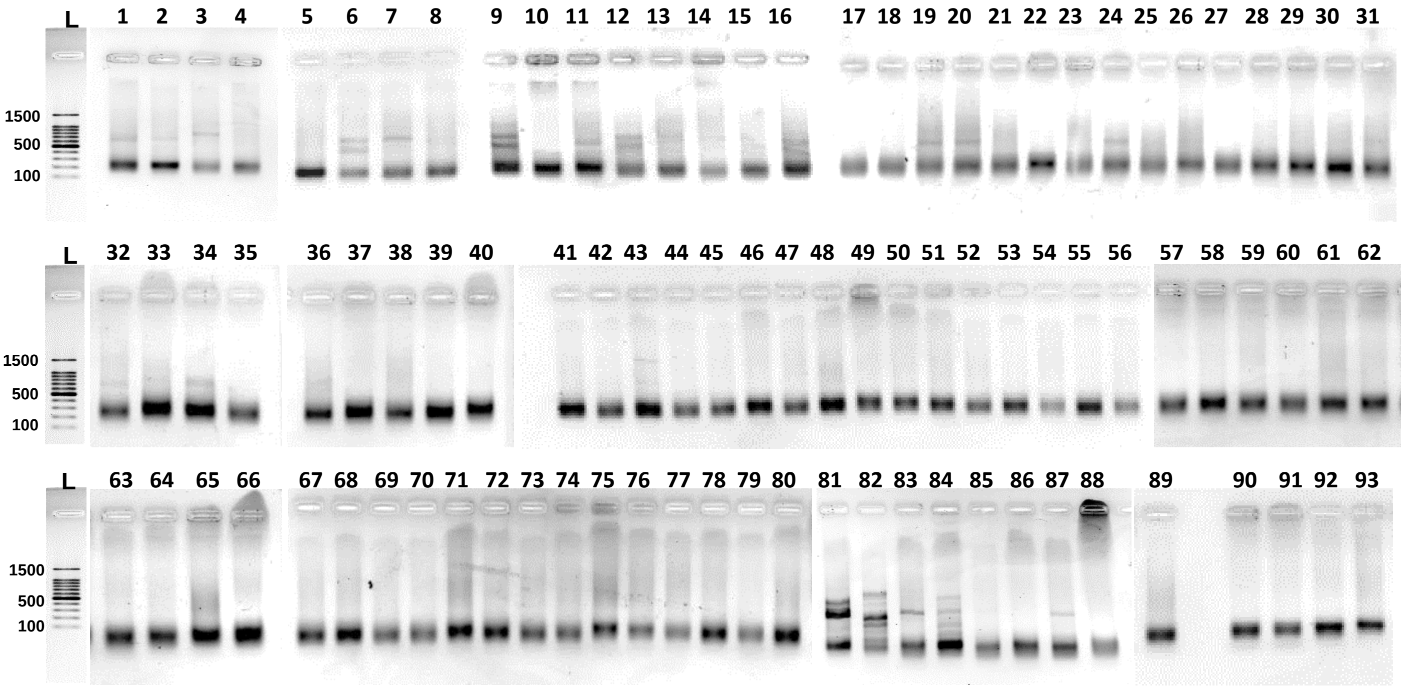
L
1 2 3 4 5 6 7 8 9 10 11 12 13 14 15 16 17 18 19 20 21 22 23 24 25 26 27 28 29 30 31
1500
500
100
L
32 33 34 35 36 37 38 39 40 41 42 43 44 45 46 47 48 49 50 51 52 53 54 55 56 57 58 59 60 61 62
1500
500
100
L
63 64 65 66 67 68 69 70 71 72 73 74 75 76 77 78 79 80 81 82 83 84 85 86 87 88 89 90 91 92 93
1500
500
100

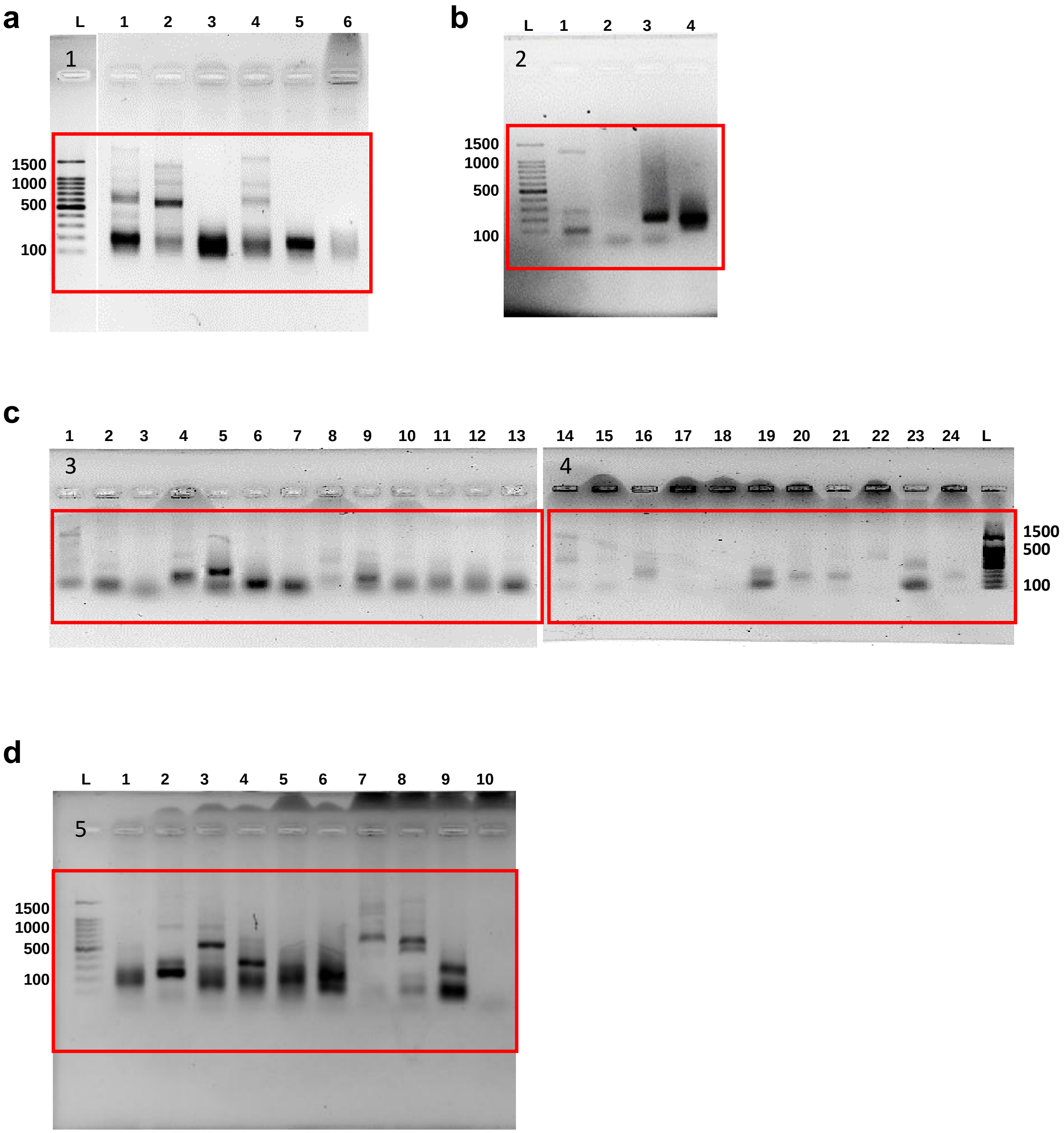

Supplementary full gel image (S6) for Figure 5: Testing the specificity of SSR primers

(a) Amplification of LafMS02 primer**.** L- 100 bp molecular weight marker 1: Elephant dung DNA (from the mucus layer) 2: Fibers taken from inside of the bolus 3: Mixture of Plant DNA (*C. zeylanicum*, *O. rhizomatis*, *S. album* and *S.*

zeylanica) 4: Fibers taken from multiple samples 5: Elephant Blood DNA (from elephant no: 66) 6: Negative control-water.

(b). Amplification of EMU14 primer. L- 100 bp molecular weight marker 1: Elephant dung DNA (from the mucus layer), 2: Fibers taken from inside of the bolus 3: Mixture of Plant DNA (*C. zeylanicum*, *O. rhizomatis*, *S. album* and *S. zeylanica*), 4: Elephant Blood DNA (from elephant no:66).

(c). Amplification of plant DNA with 24 primers. 1: EMU03, 2: EMU04, 3: EMU06, 4: EMU07, 5: EMU10, 6: EMU09, 7: EMU11, 8: EMU12, 9: EMU13, 10: EMU14, 11: EMU15, 12: EMU17, 13: LafMS02, 14: LafMS06, 15: LA2, 16: LA3, 17: LA4, 18: LA5, 19: LA6, 20: EMX-1, 21: EMX-2, 22: EMX-3, 23: EMX-4, 24: EMX-5, L- 100 bp ladder.

(d). Amplification of plant DNA with nine randomly selected primers (repeated PCR). L- 100 bp ladder 1: EMU07, 2: EMU09, 3: EMU10, 4: EMU13, 5: EMU14, 6: EMU15, 7: LafMS06, 8: EMX-4, 9:LA6 , 10: Negative control (water).

<u>Exposure times-</u>

Gel Image number	Exposure time
1	3.555
2	3.637
3	3.622
4	3.765
5	5.526

Gel images with different exposures manually changed.

Exposure 1

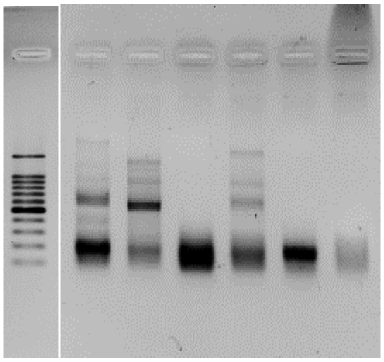

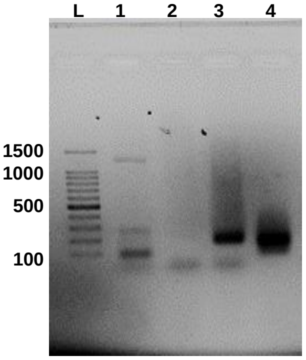

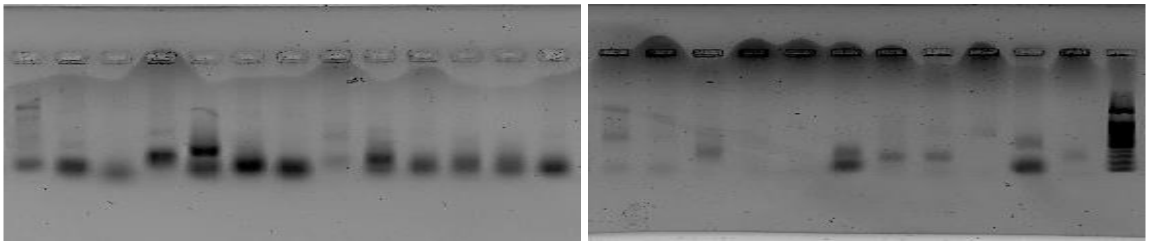

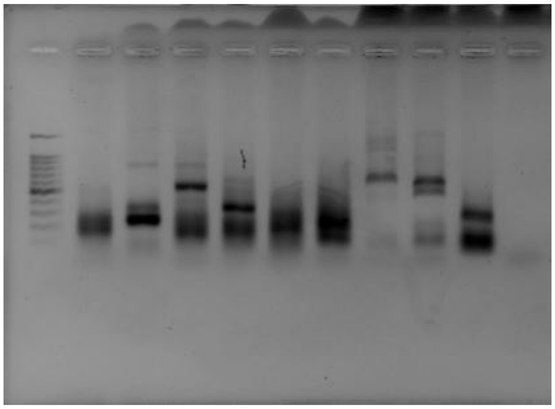

Exposure 2

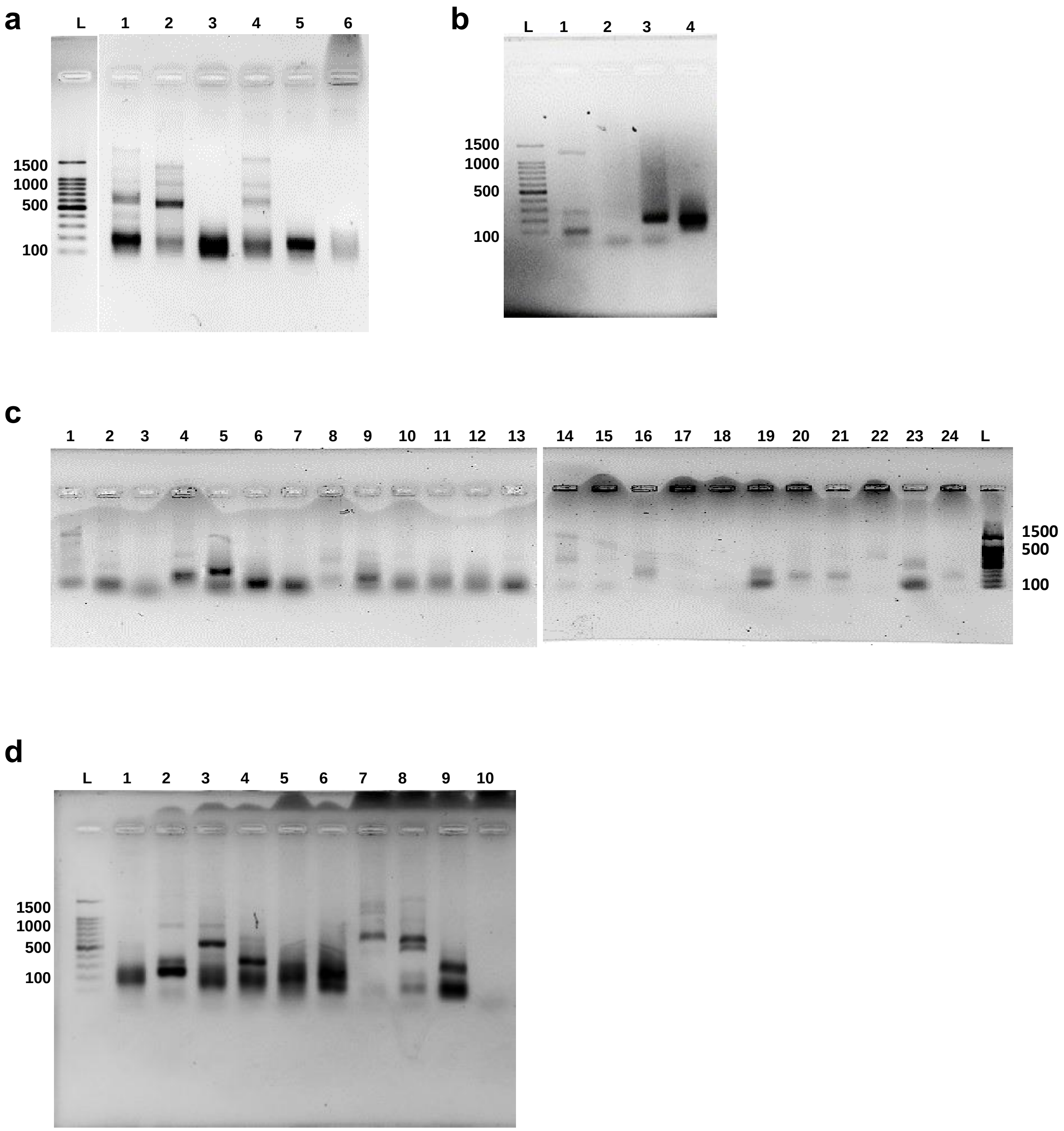
a
L 1 2 3 4 5 6
1500
1000
500
100
b
L 1 2 3 4
1500
1000
500
100
c
1 2 3 4 5 6 7 8 9 10 11 12 13
14 15 16 17 18 19 20 21 22 23 24 L
1500
500
100
d
L 1 2 3 4 5 6 7 8 9 10
1500
1000
500
100

Exposure 3

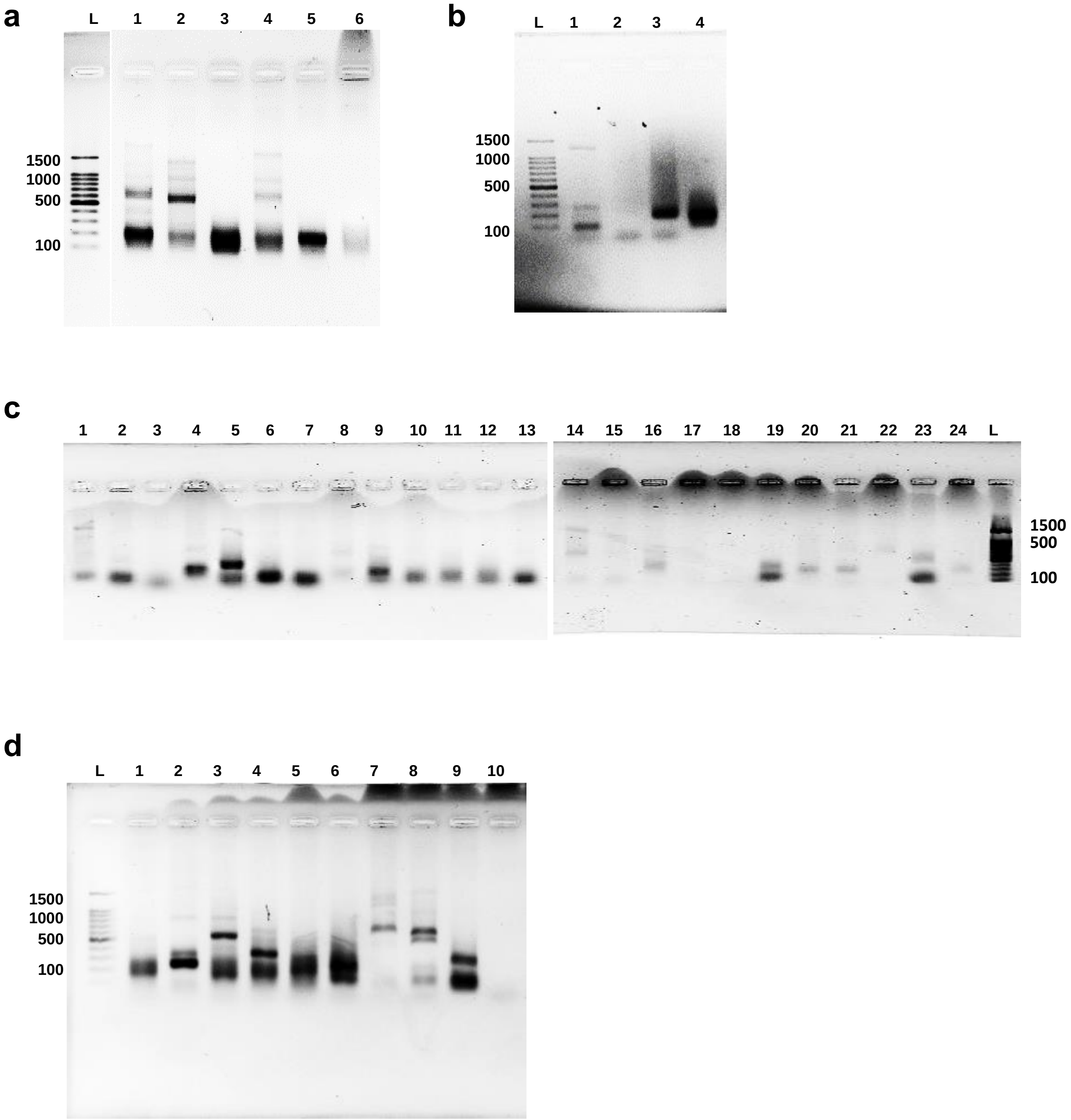
a
L 1 2 3 4 5 6
1500
1000
500
100
b
L 1 2 3 4
1500
1000
500
100
c
1 2 3 4 5 6 7 8 9 10 11 12 13
14 15 16 17 18 19 20 21 22 23 24 L
1500
500
100
d
L 1 2 3 4 5 6 7 8 9 10
1500
1000
500
100

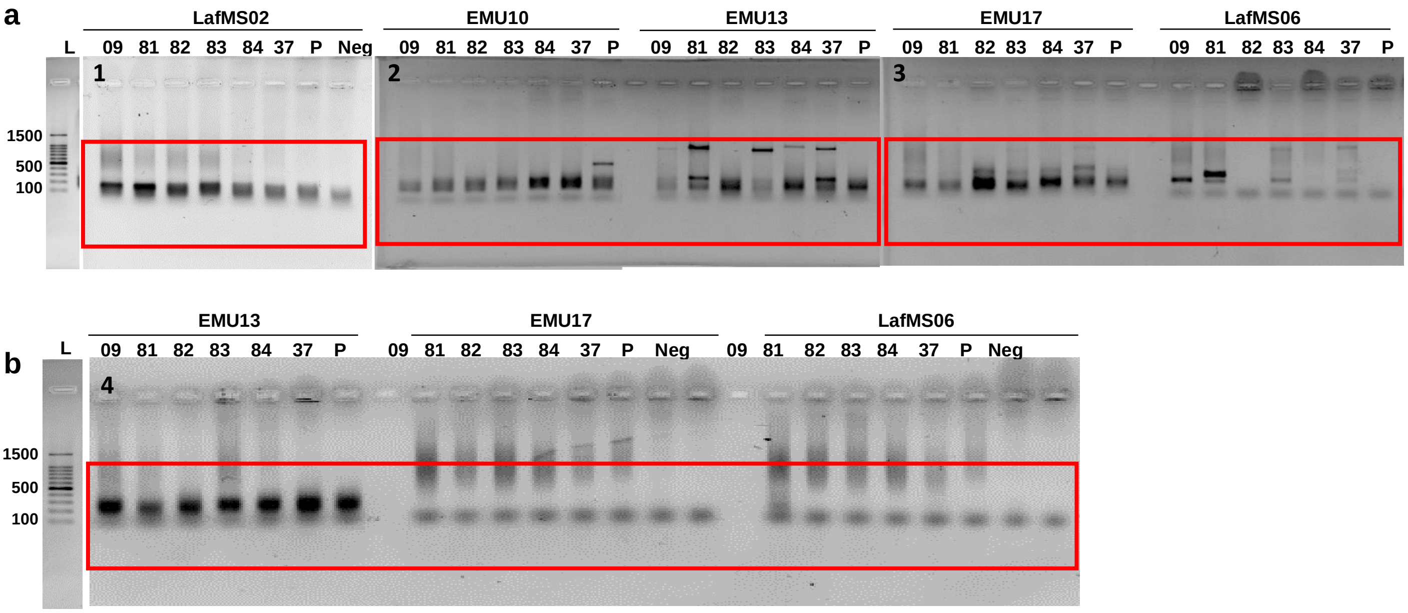

Supplementary full gel image (S7) for Figure 6: Optimization of annealing temperature

(a). Annealing temperatures 62 ºC – 58 ºC in ‘touch down 58’ PCR for randomly selected samples (09,81,82,83,37) using 05 polymorphic primers; LafMMS02, EMU10, EMU13, EMU17, LafMS06), L- 100 bp molecular weight marker (Promega, Cat no: G2101), P – Plant DNA mixture, N- Negative control (water).

(b). Annealing temperatures 69 ºC – 65 ºC in ‘touch down 65’ PCR for three primers having high annealing temperature for randomly selected samples (09,81,82,83,84,37), L- 100 bp molecular weight marker (Promega, Cat no: G2101), P – Plant DNA mixture, N- Negative control (water).

<u>Exposure times-</u>

Gel Image number	Exposure time
1	3.021
2	5.210
3	5.177
4	4.737

Gel images with different exposures manually changed.

Exposure 1

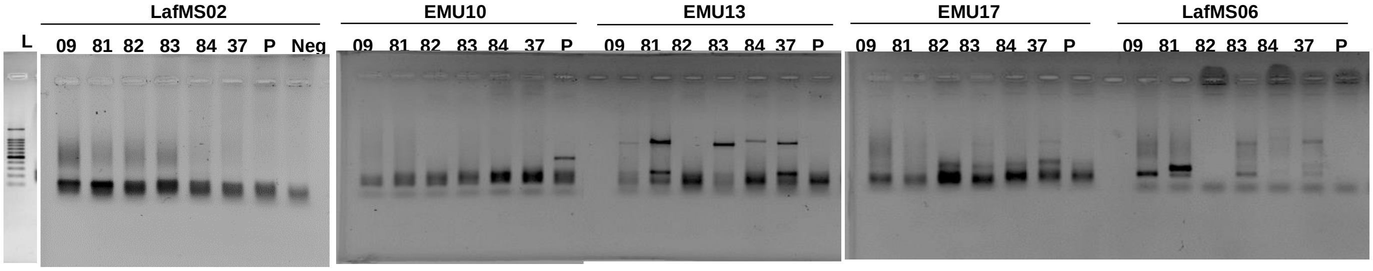

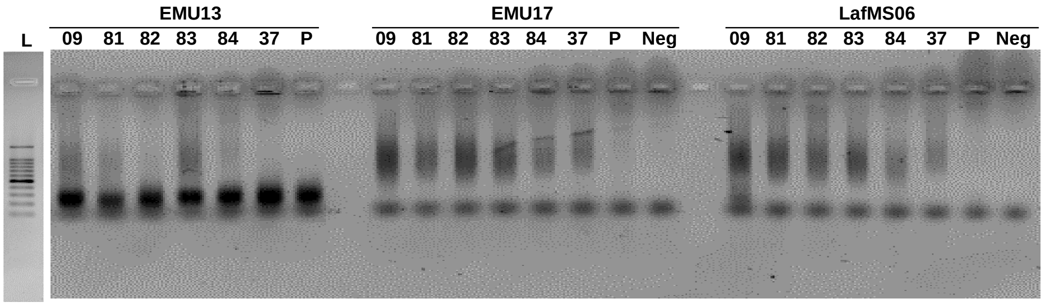

Exposure 2

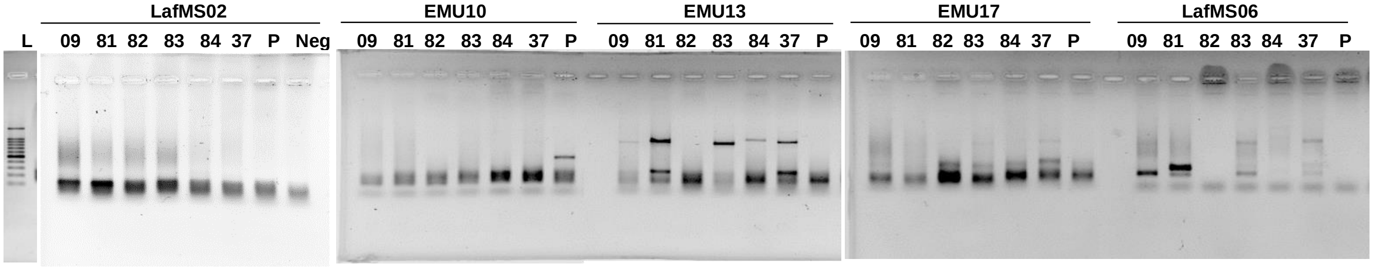

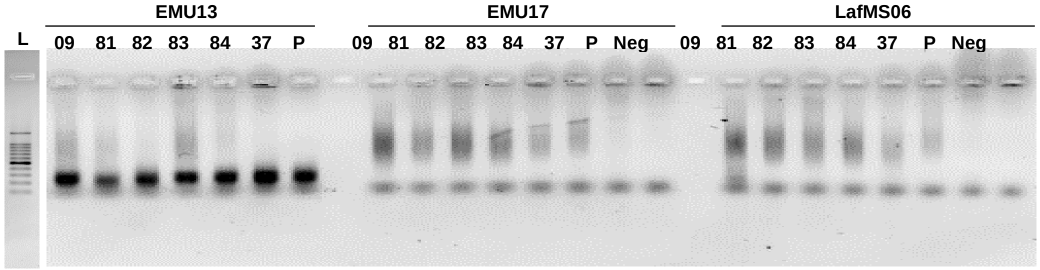

Exposure 3

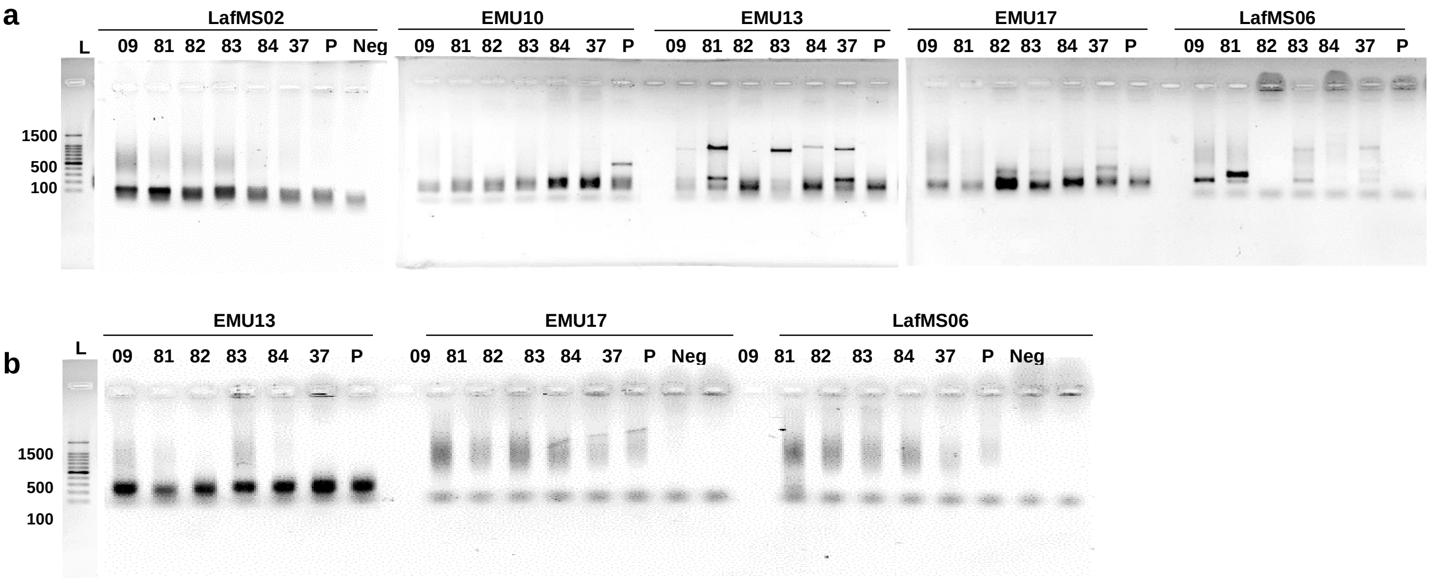

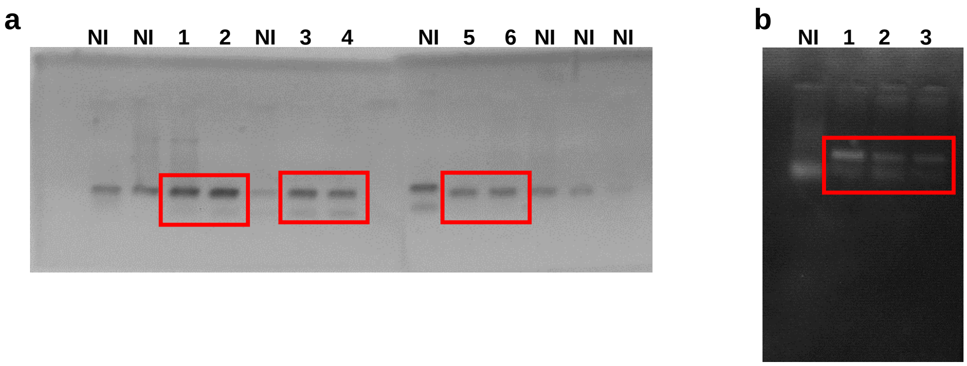

Supplementary full gel image (S8) Supplementary Figure S1: Optimization of elephant dung DNA extraction

c. DNA extraction with different incubation times and temperatures using QIAGEN QIAamp Fast DNA Stool Mini Kit (Cat. No: 51604). Subsamples were lysed using 1 mL of lysis buffer provided by the kit for different temperatures for different times while shaking at 700 rpm. The rest of the steps followed the instructions provided by the kit. The extracted DNA was amplified with EMU15 from dung DNA using ‘touchdown 55’ algorithm. (1). 56 °C Overnight incubation; (2). 56 °C 4h incubation; (3). 56 °C 3h incubation; (4). 56 °C 2h incubation; (5). 70 °C >1h incubation; (6). 70 °C 1h incubation (NI – Not included in the manuscript)

d. Effect of storage condition of dung samples. The subsamples were stored at 4 °C for 4 weeks and DNA was extracted using the QIAGEN QIAamp Fast DNA Stool Mini kit (Cat.No: 51604) during the periods of 2 weeks and 4 weeks. The DNA was quantified using NanoDrop Spectrophotometer. In order to check the quality of DNA for PCR, the extracted DNA were amplified with EMU15 using dung DNA following ‘touchdown 55’ algorithm. (1) Fresh DNA; (2) Mucus layer stored for 2 weeks; (3) Mucus layer stored for 4 weeks

Gel images with different exposures manually changed.

Exposure 1

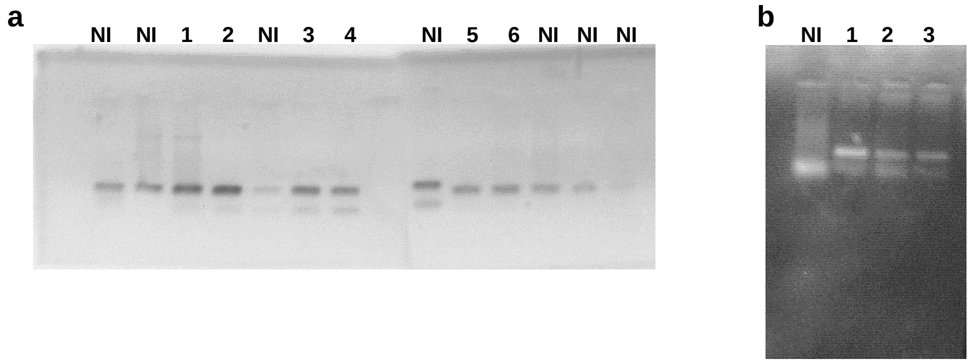

Exposure 2

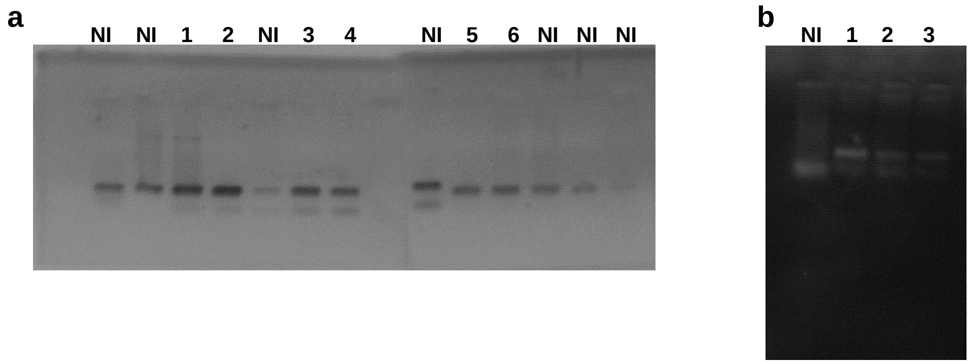

Supplement: Supplementary file 1 — Supplementary Information. [file 41598_2021_88034_MOESM1_ESM.pdf]
